# Impact of chronic social stress on molecular markers of skin regeneration during experimental excisional wounding

**DOI:** 10.3389/fimmu.2025.1656214

**Published:** 2025-12-03

**Authors:** Lyudmyla Makyeyeva, Igor Belenichev, Olena Aliyeva, Oleksandr Frolov, Pavlo Petakh, Oleksandr Kamyshnyi

**Affiliations:** 1 Department of Histology, Cytology and Embryology, Zaporizhzhia State Medical and Pharmaceutical University, Zaporizhzhia, Ukraine; 2 Department of Physiology, Immunology, and Biochemistry with a Course in Civil Protection and Medicine, Zaporizhzhia National University, Zaporizhzhia, Ukraine; 3 Department of Pharmacology and Medical Formulation with Course of Normal Physiology, Zaporizhzhia State Medical University, Zaporizhzhia, Ukraine; 4 Department of Biochemistry and Pharmacology, Uzhhorod National University, Uzhhorod, Ukraine; 5 Department of Microbiology, Virology, and Immunology, I. Horbachevsky Ternopil National Medical University, Ternopil, Ukraine

**Keywords:** wound healing, chronic social stress, apoptosis, transcription factors, skin

## Abstract

**Background:**

The second decade of the 21st century has seen increased environmental stressors, global pandemics, and armed conflicts, all contributing to heightened population morbidity and mortality. Among the affected health outcomes, wound healing has emerged as a critical physiological process vulnerable to impairment by psycho-emotional and social stress. Chronic stress is known to delay tissue repair, disrupt inflammatory responses, and exacerbate oxidative damage, yet the molecular mechanisms linking social stress to impaired skin regeneration remain insufficiently understood.

**Methods:**

This study investigated the impact of chronic social stress (CSS) on molecular pathways involved in apoptosis, cytoprotection, and proliferation during skin wound healing in a rat model. A total of 120 male Wistar rats were allocated into experimental (CSS-exposed), aggressor, and control groups based on behavioral assessments. CSS was induced by combining social isolation and continuous exposure to aggressive conspecifics for 21 days. Full-thickness excisional wounds were created, and skin samples were collected during wounding and at days 1, 3, 7, 14, and 30 post-injury to correspond with the inflammatory, proliferative, and remodeling phases of healing. Immunohistochemical analyses were performed to assess the expression of key markers: HIF1α, BCL2, caspase-3, caspase-9, NRF2, SOX2, PDGFRB, CGRP, p62, and LC3BB.

**Results:**

Chronic social stress significantly delayed wound closure and altered the expression of molecular markers critical for tissue regeneration. Immunohistochemical analysis revealed reduced expression of cytoprotective (NRF2, HIF1α) and regenerative (PDGFRB, CGRP) markers, alongside increased markers of apoptosis (caspase-3, caspase-9) and impaired autophagy (p62, LC3BB) in periwound of CSS-exposed rats tissues compared to controls. These molecular alterations corresponded with delayed progression through the inflammatory and proliferative phases and incomplete remodeling at day 30.

**Conclusion:**

The findings demonstrate that chronic social stress impairs skin wound healing by disrupting the balance of apoptosis, cytoprotection, and proliferation at the molecular level. Suppression of pro-inflammatory and regenerative pathways, combined with enhanced oxidative stress and apoptosis, underlies the observed delays in tissue repair. These results highlight the importance of addressing psycho-social factors in the management of wound healing and suggest potential molecular targets for therapeutic intervention in stress-impaired tissue regeneration.

## 1 Introduction

The second decade of the 21st century has been marked by a deterioration of environmental conditions, global pandemics, heightened solar activity, and a large-scale armed conflict in Central Europe involving all types of weaponry except nuclear arms (1, 2). All of these factors have contributed to increased population mortality not only due to the use of various types of weaponry, but also as a result of a rise in the incidence of non-communicable diseases—particularly cardiovascular, oncological, and other chronic conditions. Stress, somatic pathologies, traumatic injuries, as well as thermal and chemical burns, have resulted in a substantial number of both military personnel and civilians requiring medical treatment for skin injuries and dermatological disorders (3–7).

Wound healing is a critical physiological process essential for recovery following injuries and surgical interventions. Impaired or delayed healing increases the risk of wound infections and complications, prolongs hospitalization, exacerbates patient discomfort, and delays the return to normal daily activities (8–12). Numerous studies have demonstrated that both acute and chronic psycho-emotional stress, social-conflict-related stress, and other behavioral factors can adversely affect the wound healing process and reduce the efficacy of reparative pharmacological agents. Stressors of varying intensity and duration have been shown to significantly delay tissue repair. For example, exposure to the Trier Social Stress Test has been associated with slower recovery of the skin’s barrier function (11, 13–16). There is evidence supporting the role of socialization in the wound healing process (14, 17). Post-stressor consequences such as depression have been shown to impair tissue regeneration and increase the risk of infection-related complications (11). Stress exposure disrupts the inflammatory phase of wound healing by suppressing the production of IL-1β and TNF-α, thereby prolonging the healing process. This effect is largely attributed to elevated post-stress cortisol levels, which inhibit the expression of pro-inflammatory cytokines that are essential for initiating and sustaining an effective wound healing response (18, 19).

Another mechanism contributing to delayed wound healing following stress is the enhancement of pro-oxidative processes in the body, including increased production of reactive oxygen species (ROS), lipid peroxidation, and oxidative modification of proteins. It is well established that chronic stress can further impair wound healing through neurochemically induced ROS overproduction, accompanied by a reduction in key antioxidant enzymes such as superoxide dismutase (SOD), glutathione peroxidase 4 (GPX4), and glutathione peroxidase 1 (GPX1) (20). ROS are essential for regulating various physiological processes, including inflammation, cell proliferation, angiogenesis, granulation, and extracellular matrix formation. However, excessive ROS production following stress can lead to delayed or impaired wound healing. Elevated ROS levels disrupt the tissue growth factor-α1 (TGF-α1) signaling pathway, suppress the expression of fibroblast growth factor (FGF), and interfere with fibroblast proliferation and migration, as well as the synthesis and deposition of collagen and fibronectin. Moreover, ROS excess impairs angiogenesis by altering endothelial cell division and migration through dysregulation of vascular endothelial growth factor (VEGF) expression, thereby compromising the formation of new blood vessels and weakening regenerative mechanisms (21–23).

All of this highlights a potential link between stress—both psycho-emotional and post-traumatic—and impaired skin wound regeneration. However, a coherent concept regarding the influence of social stress on wound healing processes and the underlying molecular mechanisms remains lacking. This gap underscores the critical relevance and interest—both for fundamental medical research and clinical practice—in elucidating the precise molecular pathways through which chronic social stress inhibits wound healing.

This research aimed to assess the impact of social stress on the molecular mechanisms of apoptosis, endogenous cytoprotection, and proliferation following excisional skin injury, and to identify the key molecular targets involved in this process.

## 2 Materials and methods

### 2.1 Animals

The study was conducted on 120 male Wistar rats, weighing 210–230 g, aged 12–13 months. The animals were obtained from the breeding facility of the Veterinary Medicine Association, LLC “Biomodelservice,” Kyiv, Ukraine. Prior to the experiment, each animal was examined by a qualified veterinarian to assess its health status. Subsequently, the animals underwent a 10-day quarantine period.

The rats were housed in polycarbonate cages measuring 550 × 320 × 180 mm, covered with galvanized steel lids sized 660 × 370 × 140 mm, and equipped with glass drinking bottles. No more than five rats were kept in each cage. Each cage was labeled with an identification number and placed on racks corresponding to these labels.

Environmental conditions in the animal room were maintained as follows: temperature 20–24°C, humidity 30–70%, and a 12-hour light/12-hour dark cycle. All rats were provided with ad libitum access to a standard laboratory diet supplied by “Phoenix” (Ukraine). Drinking water was given without restriction and sourced from the municipal water supply after reverse osmosis and UV sterilization. Alder (Alnus glutinosa) wood shavings, autoclaved prior to use, served as bedding material.

Following wound induction, animals were housed individually until complete healing was observed.

All procedures involving animals were conducted in accordance with the “International Guidelines for Biomedical Research Involving Animals”. Additionally, compliance was ensured with the main provisions of the “Rules for Conducting Work Using Experimental Animals” approved by the Ministry of Health of Ukraine (Order No. 753 dated August 12, 1997), the European Convention for the Protection of Vertebrate Animals Used for Experimental and Other Scientific Purposes (Strasbourg, March 18, 1986), Ministry of Health of Ukraine Order No. 281 dated November 1, 2000, “On Measures for Further Improvement of Organizational Norms for Work with Experimental Animals,” and the Law of Ukraine No. 3447-IV “On the Protection of Animals from Cruel Treatment” (February 21, 2006, as amended December 9, 2015, basis 766-19). The experiment was approved by the Bioethics Commission of Zaporizhzhia National University (protocol No. 7 dated February 27, 2025).

### 2.2 Experimental model of chronic social stress

Chronic social stress (CSS) was induced in the experimental group, which was preselected for high stress susceptibility based on behavioral parameters in the open field test (increased immobility, reduced exploratory activity). This group included 30 male rats (n=6 per wound healing time point).

To model CSS, animals were subjected to a validated protocol combining social isolation and continuous psychoemotional stress over a 21-day period. Each rat was individually housed to eliminate social contact (social deprivation), while being continuously exposed to a threatening social environment. Specifically, four aggressive conspecifics—selected based on high aggression indices from the open field —were housed in adjacent transparent cages (one on each side of the isolated rat). The cages allowed visual, auditory, and olfactory contact, but prevented physical interaction, thereby maintaining constant social pressure and threat perception without direct physical harm.

Aggressor rats were rotated every 3–4 days to prevent habituation and to sustain unpredictability and psychological strain. The experimental environment was kept under standard laboratory conditions. The aggressor group consisted of dominant adult male rats that had been preselected based on their stable aggressive behavior in repeated social interaction tests. These animals were housed individually for at least two weeks prior to the experiment to establish territorial dominance. The aggressor rats were not subjected to any stress procedures themselves and were excluded from subsequent behavioral and biochemical analyses.

This protocol is based on and adapted from previously published CSS paradigms known to reliably induce depressive-like states and delayed wound healing in rodents (24–29). The presence of stress was confirmed using the “Open Field” method.

### 2.3 “Open Field” method

Stress was confirmed using the “Open Field” method, which was conducted on all animals before and after the induction of chronic social stress.

Animal behavior was observed in an open field apparatus measuring 80 × 80 cm, divided into 10 × 10 cm squares, over a 5-minute period. The field was illuminated by a 100 W light source positioned 1 meter above the surface. Each animal was placed in the center of the field, and the following parameters were recorded: number of urinations and defecations, freezing episodes, latency (seconds) to the first exit from the central square, number of crossed central and peripheral squares, duration (seconds) and frequency of grooming behaviors (both long and short bouts), and number of rearings on hind legs during the 5-minute test.

After testing each animal, the surface of the open field was thoroughly cleaned with water and dried.

### 2.4 Group allocation of animals

Based on the results of the “Open Field” Test, 43 males exhibiting higher aggression levels were assigned to the aggressor group. Thirty males showing greater susceptibility to stress were allocated to the experimental group. From the remaining animals, 20 males were assigned to the control group.

### 2.5 Preparation of biological material

Skin tissue excision was performed between the shoulder blades of the animals to create a full-thickness wound under thiopental anasthesia (40mg/kg, Kyivmedpreparat). The full-thickness wound model (involving the epidermis, dermis, and subcutaneous tissue) was established as follows: after aseptic preparation and anesthesia, a circular excision measuring 1–1.3 cm in diameter was made in the interscapular region on the shaved dorsal surface of the rats (30).

Tissue samples from the structural components of the wound bed, as well as from adjacent intact skin (within 1 cm of the wound margin), were collected on days 1, 3, 7, 14, and 30 after injury. In this study, special attention was given to the periwound area, defined as tissue located up to 5 mm beyond the wound edge. After material collection at each time point, the animals were withdrawn from the experiment.

According to modern wound healing periodization, days 1 and 3 correspond to the inflammatory phase, days 7–14 to the proliferative phase, and day 30 to the re-modeling phase.

Following the creation of the excisional wound, animals were housed individually, and bedding was replaced weekly under aseptic conditions. No signs of bacterial contamination or infection were observed in either group throughout the study period.

In order to determine the concentration of Slc6a4 used a Rat Slc6a4 (Sodium-Dependent Serotonin Transporter) ELISA Kit (Catalogue No. MBS762454, MyBioSource, USA) in the cytosolic fraction of brain homogenates. Blood from rats in control and experimental groups (n=10) after stressing was rapidly removed from the brain, which was then carefully separated from the meninges. The selected brain regions were immediately placed in liquid nitrogen, ground to a fine powder, and homogenized in a tenfold volume of medium (at +2°C) containing (in mmol): 250 mM sucrose, 20 mM Tris-HCl buffer, and 1 mM EDTA (pH 7.4). The cytosolic and mitochondrial fractions were obtained by differential centrifugation at +4°C using a Sigma 3-30k refrigerated centrifuge (Germany). To remove large cellular debris, the homogenate was first centrifuged for 7 min at 1000 × g; the resulting supernatant was then centrifuged again for 20 min at 17 000 × g.

### 2.6 Tissue preparation and immunohistochemical staining

The obtained skin samples were fixed in a 10% solution of neutral formalin in a vessel with darkened glass, stored at room temperature for 3 days before histological experiments. Then, according to the standard histological technique, the skin was placed in paraffin blocks, from which serial microtome sections with a thickness of 5 μm were made. Serial sections were made using a Thermo Scientific HM 325 microtome (Thermo Scientific, Massachusetts, USA) and mounted onto positively charged glass slides. Following deparaffinization and rehydration through graded alcohols and xylene, antigen retrieval was performed using a PT module according to the manufacturer’s instructions.

After retrieval, tissue sections were rinsed in distilled water for two changes of 5 minutes each, followed by incubation in TRIS buffer for 5 minutes. Endogenous peroxidase activity was blocked by incubating the sections in 3% hydrogen peroxide (prepared by diluting 6% H_2_O_2_ in distilled water) for 20 minutes at room temperature. Slides were then rinsed in distilled water (2 × 5 minutes) and incubated again in TRIS buffer for 5 minutes.

To reduce non-specific binding, a protein blocking solution was applied for 5 minutes, followed by a TRIS buffer rinse for 5 minutes. Primary antibodies, diluted appropriately, were applied to the tissue sections and incubated in a humidified chamber at room temperature for 30 minutes. After a 5-minute TRIS buffer wash, slides were incubated with a biotinylated secondary antibody (appropriately diluted) for 10 minutes at room temperature.

Following another 5-minute TRIS wash, sections were incubated with an HRP-conjugated polymer detection reagent for 10 minutes. Slides were then washed in TRIS buffer for 5 minutes.

For chromogenic detection, DAB staining was performed using DAB 0.73 reagent diluted in 1 mL phosphate-buffered saline (PBS). The DAB solution was applied to the tissue sections, and color development was monitored for up to 5 minutes, stopping when optimal staining intensity was achieved.

Sections were rinsed in distilled water and counterstained with Mayer’s hematoxylin. Differentiation was carried out using ammonium solution (1:25 dilution), followed by a final rinse in distilled water. Slides were dehydrated through an ethanol gradient (95%, 95%, 100%, 100%; 5 minutes each), air-dried, cleared in three changes of xylene, and coverslipped with a permanent mounting medium.

To evaluate endogenous cytoprotection, the intensity of HIF1α expression was assessed in skin sections (epidermis and dermis) using primary antibodies (Santa Cruz Biotechnology, USA, Cat. No. sc-13515) and secondary antibodies (antibodies-online, Germany, Cat. No. ABIN7205196).

For the assessment of apoptosis, the expression of the antiapoptotic protein BCL2 (Santa Cruz Biotechnology, USA, Cat. No. sc-7382), as well as the expression of caspase-3 and caspase-9 (Santa Cruz Biotechnology, USA, Cat. Nos. sc-65497 and sc-56076, respectively) was analyzed in the epidermis and dermis. All sections were incubated with the same secondary antibody (antibodies-online, Germany, Cat. No. ABIN7205196).

As a marker of endogenous cytoprotection, NRF2 expression was evaluated (Thermo Fisher Scientific, USA, Cat. No. PA5-27882) with secondary antibodies (anti-bodies-online, Germany, Cat. No. ABIN2690458). NRF2 is a transcription factor that regulates cellular defense mechanisms against toxic and oxidative damage by modulating the expression of genes involved in oxidative stress response and drug detoxification.

To assess the differentiation of skin cell lineages, SOX2 expression was evaluated (Abcam, USA, Cat. No. ab97959) using the same secondary antibodies (ABIN2690458).

For evaluation of skin regeneration, PDGFRβ expression was analyzed (Invitrogen, USA, Cat. No. MA5-15143) along with secondary antibodies (ABIN2690458). PDGFRβ encodes a member of the platelet-derived growth factor (PDGF) and vascular endothelial growth factor (VEGF) receptor families. It plays a critical role in vascular development, and its deficiency impairs tissue integrity and function in multiple organs, including the brain, heart, kidneys, skin, and eyes.

Skin regeneration was also assessed by measuring CGRP (Calcitonin Gene-Related Peptide) expression (Synaptic Systems GmbH, Germany, Cat. No. 414004) with corresponding secondary antibodies (antibodies-online, Germany, Cat. No. ABIN2690462). CGRP is a potent vasodilator that contributes to protective mechanisms relevant to both physiological and pathological wound healing.

To evaluate autophagy, expression levels of nuclear pore glycoprotein p62 (Santa Cruz Biotechnology, USA, Cat. No. sc-48402) and LC3BB (Novus Biologicals, USA, Cat. No. NB100-2220H) with corresponding secondary antibodies (antibodies-online, Germany, Cat. No. ABIN7205196 for p62 and ABIN2690458 for LC3BB).

Images were acquired using the ZEISS Axioscan 7 (ZEISS, Germany) and analyzed with QuPath software (version 0.4.4, University of Edinburgh, UK). For all listed markers (HIF1α, BCL2, caspase-3, caspase-9, NRF2, SOX2, PDGFRB, CGRP, p62, LC3BB), quantitative analysis of DAB signal intensity was performed by calculating the percentage of positively stained cells—i.e., cells with strong marker expression of all nucleated cells.

### 2.7 Statistical analysis

Statistical analysis and presentation of the experimental results were performed using the IBM SPSS Statistics software, version 26 (IBM Corp., Armonk, NY, USA). In each group of the obtained quantitative indicators, an analysis of the normality of the distribution was carried out using the one-sample Kolmogorov-Smirnov test. The Student’s t-test was used to obtain statistical conclusions when comparing samples of variables. The Bonferroni correction was applied. A difference was considered statistically significant at p ≤ 0.05. Correlation analyses were performed using Pearson’s correlation coefficient. Statistical significance was set at p < 0.05.

Generalized data from the study of morphological features of rat skin during wound healing were expressed as the arithmetic mean and its standard deviation (mean ± SD).

## 3 Results

Rats exposed to the CSS exhibited clear behavioral and neurochemical signs of chronic stress In the group of animals exposed to chronic social stress, impaired cognitive activity was observed—manifested by a reduction in overall activity and total distance traveled—alongside increased anxiety, indicated by decreased entries into the illuminated center, reduced frequency and duration of grooming behaviors, and increased defecations and urinations as well as freezing, compared to the non-stressed control group. (**Supplementary Figure 1**). At the biochemical level, the cytosolic concentration of the serotonin transporter (Slc6a4) in brain homogenates was markedly reduced in stressed rats (392.76 ± 16.32 pg/mL) compared with controls (796.84 ± 13.67 pg/mL, p < 0.0001). Correlation analysis revealed a strong negative relationship between Slc6a4 levels and latency to enter the light zone (r = –0.90, p < 0.001), indicating that animals with lower serotonergic transporter expression displayed higher anxiety and behavioral inhibition. These data confirm that chronic social stress effectively induced a pronounced stress response in rats, manifested by increased anxiety-like behavior and a significant reduction in serotonergic transporter expression.

Behavioral observations across both the stressed and control groups during various wound healing time points revealed the following: in the group subjected to chronic social stress, a marked increase in caspase-3 expression was observed. Caspase-3 is known to play a key role in the induction of apoptosis in neutrophils, macro-phages, and fibroblasts during skin wound healing. Notably, baseline caspase-3 expression in the dermis of rats that experienced stress prior to injury was already elevated compared to the control group.

In all tissues of the post-stress group, caspase-3 expression increased significantly between day 1 and day 3 post-wounding, exceeding that observed in the control group. In contrast, in the control animals, caspase-3 expression progressively decreased from day 7 and reached minimal levels by day 14. However, in the stressed group, caspase-3 expression remained elevated on days 7 and 14, and only declined by day 30 post-injury (**Supplementary Figures 2, 3**).

Our study revealed an increased expression of caspase-9 in the skin tissues of both experimental groups. It is worth noting that the baseline levels of caspase-9 in the stressed rats (prior to wounding) were significantly higher than those in the control group. Following wound infliction, a marked elevation in caspase-9 expression was observed from day 1 through day 7 post-injury across all skin layers. Pre-exposure to stress had a potentiating effect on caspase-9 expression. The most pronounced changes in caspase expression were detected in the dermis, highlighting the need for further investigation to elucidate this phenomenon.

In addition, we found a suppression of the expression of the anti-apoptotic protein Bcl-2 in all skin tissues during the wound healing period. Baseline Bcl-2 levels were lower in the stress-exposed rats, with the most significant differences between the stressed and control groups also observed in the dermis. Wound infliction and the subsequent healing process occurred along with reduced Bcl-2 expression from day 1 to day 30, particularly in the stressed group. The dermis was the most affected by Bcl-2 deficiency.

These findings indicate apoptosis activation in all three analyzed skin layers following mechanical injury. Chronic social stress appears to create an unfavorable molecular and metabolic background, promoting the initiation and progression of apoptotic processes in skin tissues after wounding.

Particular attention should be given to changes in the expression of endogenous cytoprotection markers during the wound healing process, especially under conditions of prior stress exposure. Notably, the results concerning the expression dynamics of hypoxia-inducible factor 1-alpha (HIF-1α) were of significant interest. It was found that chronic social stress resulted in a marked suppression of HIF-1α expression across all skin layers (**Supplementary Figures 4, 5**), with the most pronounced decrease observed in the dermis.

Wound infliction led to divergent patterns of HIF-1α expression between the experimental groups. In the control group, HIF-1α expression increased one day after wounding, followed by normalization by day 3 of the observation period. In contrast, the stress-exposed group exhibited a markedly different pattern, characterized by a sustained and significant decrease in HIF-1α expression (threefold reduction in the dermis), persisting up to day 14 post-injury.

Similarly, expression of the transcription factor SOX2, which plays an important role in endogenous cytoprotection and wound repair, was also lower in the stress-exposed group compared to controls across all skin tissues. Mechanical injury induced varying patterns of SOX2 expression during the healing process. In the control group, SOX2 expression increased in all skin tissues as early as day 1 post-injury, returning to baseline levels by day 7. In contrast, pre-exposure to stress was associated with a consistent downregulation of SOX2 expression from day 1 to day 14, particularly in the dermal layer.

As shown in **Supplementary Figure 6**, prior stress exposure led to a reduction in the expression of platelet-derived growth factor receptor beta (PDGFRβ) across all skin tissues when compared to the control group (**Supplementary Figure 7**). In the stressed group, Slc6a4 exhibited a strong positive correlation with PDGFRβ (r = 0.896, p = 0.00045), indicating that platelet-derived growth factor receptor beta may serve as a key marker of wound healing. PDGFRβ plays a critical role in angiogenesis and fibroblast activation, processes essential for tissue repair and regeneration. Wound infliction further suppressed PDGFRβ expression in both control and stressed animals. However, the degree of PDGFRβ downregulation was significantly greater in the stress-exposed group at all corresponding time points. Notably, this suppression persisted up to day 30 post-injury. These findings indicate that chronic stress inhibits PDGFRβ and its associated signaling functions during the early phases of wound healing.

A similar pattern was observed with the expression of calcitonin gene-related peptide (CGRP), which plays a pro-reparative role during tissue regeneration. In rats subjected to chronic social stress, CGRP expression was significantly lower than in controls across all skin tissues. While wound infliction in the control group led to a transient decrease in CGRP expression from day 1 to day 14, the stress group exhibited a more pronounced and prolonged suppression lasting through day 30. This indicates impaired wound healing capacity, as CGRP is known to stimulate vascular endothelial growth factor (VEGF), thereby promoting neovascularization in the injured area. Additionally, CGRP appears to support keratinocyte and fibroblast proliferation.

Expression of the microtubule-associated protein 1 light chain 3B (LC3B), a marker of autophagosomes, was also affected by both stress and wounding. In animals exposed to chronic social stress, LC3B levels in skin tissues were significantly lower than in the control group. During the wound healing period, especially in the early inflammatory phase, LC3B expression remained lower in the stressed group. In contrast, the control group showed a significant increase in LC3B expression shortly after wounding, consistent with established views on the role of autophagy in wound healing phases.

Chronic stress also led to a significant reduction in the expression of the nuclear pore glycoprotein p62 in all skin tissues compared to controls. This protein plays a crucial role in the SIRT1/Nrf2/p62 signaling pathway and is implicated in wound healing and autophagic regulation. In the control group, p62 expression increased from day 1 to day 7 post-injury, suggesting activation of reparative and adaptive tissue responses during this period. In contrast, stress-exposed animals exhibited a dysregulated p62 response—initial suppression from day 1 to day 7, followed by a delayed increase between days 14 and 30. This may reflect either delayed tissue repair following stress or the activation of alternative pathways, such as ferroptosis.

Similar results were obtained for nuclear factor erythroid 2–related factor 2 (NRF2), another key component of the SIRT1/Nrf2/p62 axis. Chronic social stress significantly downregulated NRF2 expression compared to controls. In the control group, wound infliction led to increased NRF2 expression from day 1 to day 7 across all skin layers, indicating activation of reparative regenerative mechanisms. In contrast, NRF2 expression in the stress group remained at baseline levels, suggesting that the SIRT1/Nrf2/p62 pathway was not activated.

Taken together, these findings indicate that chronic social stress exerts a negative influence on reparative skin regeneration following mechanical injury by enhancing apoptosis and suppressing the expression of transcriptional and cytoprotective defense factors.

## 4 Discussion

The chronic social stress model selected in our study adequately reflects the clinical manifestations of social stress observed in humans. Social stress is a form of psychological stress that arises from exposure to stressors of social origin and manifests through continuous or recurrent interpersonal interactions (such as prolonged deployment in combat zones, bullying, sexual humiliation, racism, or abusive relationships). These stressors lead to fatigue, sleep disturbances, and various health problems (31).

Chronic social stress can induce a state of low-grade, persistent inflammation, immune suppression, and stable neurochemical alterations—particularly within the serotonergic and dopaminergic systems (32). Considering the close interplay between dopaminergic and serotonergic transmission in regulating releasing systems, such stress may result in sustained disturbances of the molecular mechanisms governing reparative regeneration, vascular tone, endothelial function, synaptogenesis, and neuropeptide synthesis (33).

Over time, these alterations lead to multiple clinical consequences, including an increased risk of cardiovascular diseases, psychiatric disorders such as depression and anxiety, and cognitive impairments, as well as a marked delay in wound healing and reduced responsiveness to reparative therapies (34, 35).

Wound healing is an orderly process that begins in a predictable manner following any tissue injury. In healthy individuals, healing progresses sequentially through three overlapping phases (36–38). There is an initial inflammatory phase, which includes hemostasis (blood clotting) and the migration of inflammatory cells into the wound. This is followed by the proliferative phase, characterized by the migration and proliferation of keratinocytes, fibroblasts, and endothelial cells, leading to re-epithelialization, neovascularization, and granulation tissue formation. The final and prolonged remodeling phase involves maturation of the extracellular matrix, aimed at restoring the structural and functional integrity of the tissue (39–41).

Success in the later phases of wound healing largely depends on the proper progression of the earlier stages. The inflammatory phase encompasses three critical components that require the recruitment of cells from the circulation: passive platelet aggregation for hemostasis, influx of neutrophils for infection control, and accumulation of macrophages to initiate tissue repair (15, 42–44). Cytokines such as plate-let-derived growth factor (PDGF), transforming growth factor-alpha (TGF-α), trans-forming growth factor-beta (TGF-β), epidermal growth factor (EGF), basic fibroblast growth factor (bFGF), and vascular endothelial growth factor (VEGF) are released into the wound bed (45, 46). In response to the influx of cytokines, leukocytes and fibroblasts produce additional cytokines, such as TNF-α and interleukin-1 beta (IL-1β) (47–49). During early inflammation, neutrophils arrive at the wound site shortly after injury, followed by macrophages and monocytes. Although both neutrophils and monocytes are recruited to the injured tissue simultaneously, neutrophils predominate due to their high abundance in the circulation. Both cell populations are attracted to the wound by a variety of inflammatory chemokines and cytokines released from the blood clot and damaged cells at the wound margins. Among their many functions, these cells are phagocytic and are responsible for clearing bacteria from the wound (50, 51).

**Figure 1:**
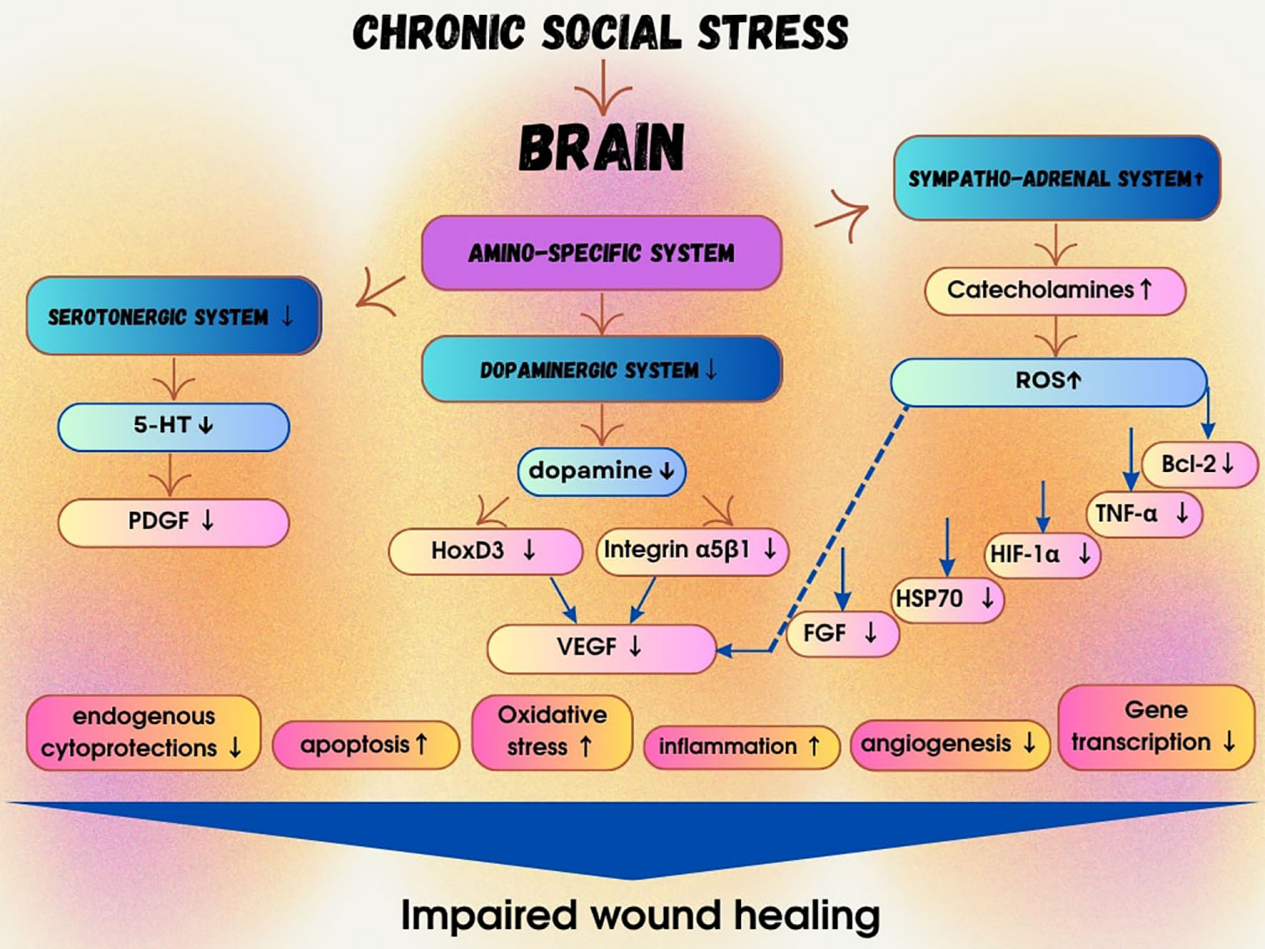
Impact of chronic social stress on molecular mechanisms leading to impaired reparative skin regeneration after excisional wounding. The results obtained revealed the detrimental effects of chronic social stress on the cutaneous wound healing process. Evidently, neurochemical, hormonal, and other disturbances mediated by social stress contribute to the enhancement and prolongation of apoptosis, suppression of transcriptional activity, and decreased expression of endogenous cytoprotective factors—ultimately impairing the healing of mechanically induced skin injuries. Stress-induced activation of the sympatho-adrenal system leads to increased production of reactive oxygen species (ROS). ROS disrupt the TGF-α1 signaling pathway, downregulate the expression of critical growth factors such as FGF, HIF-1α, HSP70, and VEGF, impair angiogenesis, inhibit bcl-2 expression, and promote uncontrolled apoptosis. Chronic social stress is also associated with dopamine deficiency, which may further reduce VEGF expression, angiogenesis, and the overall wound healing capacity. Moreover, chronic social stress results in serotonin depletion, which is linked to reduced expression of connective tissue growth factor (CTGF) and platelet-derived growth factor (PDGF), further delaying skin regeneration and enhancing apoptosis. These findings are of fundamental significance, as they elucidate the molecular mechanisms underlying wound healing and highlight potential novel targets for pharmacological modulation of reparative skin regeneration following injury.

Furthermore, the newly recruited monocytes, which differentiate into macrophages upon entering the tissue parenchyma, begin to initiate tissue repair. For example, macrophages produce enzymes such as hyaluronidase, elastase, and collagenase, which degrade hyaluronic acid, elastin, and collagen in the connective tissue (52, 53). Macrophages weaken the extracellular matrix to facilitate the migration and proliferation of fibroblasts, keratinocytes, and endothelial cells, which form new tissue during the subsequent proliferative phase of healing. In addition to preparing the extracellular matrix for tissue growth, macrophages also synthesize and release multiple growth and regulatory factors that are crucial for coordinating new tissue formation (54, 55). Approximately four days after injury, macrophages, fibroblasts, and blood vessels infiltrate the stroma, forming granulation tissue (56). Macrophages stimulate fibroplasia and angiogenesis by releasing growth factors; fibroblasts provide structural support by synthesizing the extracellular matrix, while blood vessels transport nutrients to the site (57). Neovascularization is mediated by a range of chemical inducers, including fibroblast growth factor 1 (FGF-1), PDGF, TGF-α, TGF-β, and VEGF (58). In addition to growth factors, the recruitment of endothelial cells to the wound also requires fibronectin deposition by nearby microvascular endothelial cells (59) (**Figure 1**).

Approximately two weeks after injury, fibroblasts undergo phenotypic transformation into myofibroblasts and migrate into the wound bed, where they begin to con-tract the wound (60). Once macrophages begin producing these growth factors, the reparative phase of healing effectively begins. Specifically, the formation of new capillaries from existing blood vessels (angiogenesis) and the deposition of new extracellular matrix by tissue fibroblasts are characteristic features of the proliferative phase of repair (61, 62). The first two phases of healing can be completed within just 12 to 14 days, whereas the final phase may continue for weeks, months, or even years after the injury (63, 64).

The role of glucocorticoids and cytokines—such as IL-8, IL-1α, IL-1β, IL-6, TNF-α, and IL-10—in wound healing processes is well established (38). Additional cytokines, chemokines, and growth factors are essential for effective wound healing. These include CXC chemokine ligand 1 (CXCL1), CC chemokine ligand 2 (CCL2), granulocyte-macrophage colony-stimulating factor (GM-CSF), monocyte chemoattractant protein-1 (MCP-1), macrophage inflammatory protein-1 alpha (MIP-1α), VEGF, TGF-β, keratinocyte growth factor (KGF), PDGF, and basic fibroblast growth factor (bFGF). Particularly important are the epidermal growth factor (EGF) family, TGF-β family, the fibroblast growth factor (FGF) family, vascular endothelial growth factor (VEGF), granulocyte-macrophage colony-stimulating factor (GM-CSF), PDGF, connective tissue growth factor (CTGF), the interleukin (IL) family, and TNF-α family. Currently, three growth factors are used clinically for wound treatment: PDGF-BB, bFGF, and GM-CSF (63, 65, 66).

Autophagy plays a critical role in various phases of wound healing. In particular, during the inflammatory phase, autophagy exerts antimicrobial effects and negatively regulates the inflammatory response, thereby preventing excessive inflammation from causing additional tissue damage (67).

During the proliferative phase, local hypoxia within the wound can induce autophagy, which plays roles in both anti- and pro-apoptotic processes, mitigates oxidative stress, and supports cell survival. Once new tissue has been generated to fill the void left by the initial injury, the final stage of wound healing begins. This phase involves tissue contraction and remodeling and is essential for the partial restoration of the original tissue structure and function. The concluding stage of wound healing—maturation—is characterized by the regression of granulation tissue and fibroplasia (68–70). This is a gradual process that may take many months to complete. During this stage, the epidermis regenerates and undergoes a reduction in transient hyper-trophy, while the temporary matrix is replaced by a dermal collagen matrix and eventually by a scar with low cellularity. Degradation of the collagen matrix is mediated by matrix metalloproteinases (MMPs), which are secreted by epidermal cells, fibroblasts, endothelial cells, and macrophages. Ultimately, the wound is replaced by newly formed functional tissue (71). Autophagy in vascular endothelial cells promotes wound angiogenesis, while autophagy in keratinocytes supports their differentiation, proliferation, and migration, thereby facilitating the completion of wound re-epithelialization. During the remodeling phase, fibroblast autophagy influences the formation of hypertrophic scars. Moreover, refractory diabetic wounds may be associated with elevated levels of autophagy, and modulation of autophagy in mesenchymal stem cells may enhance their therapeutic potential for wound healing (72–74).

The role of HSPs in wound healing is well established. HSP70 accelerates wound closure by enhancing macrophage-mediated phagocytosis of wound debris. Disruption of HSP70-mediated enhancement of phagocytosis abolishes the HSP-mediated acceleration of the healing process. HSP70 also stimulates endogenous cytoprotective mechanisms, supports energy supply for reparative regeneration processes, and pro-longs the half-life of HIF-1 (75, 76). HIF-1, a key regulator of oxygen homeostasis, plays a crucial role in determining wound healing outcomes. HIF-1 is involved in all stages of wound healing due to its functions in cell migration, survival under hypoxic conditions, cell proliferation, growth factor release, and matrix synthesis throughout the healing process. Activation of HIF-1 is also a major driver of angiogenesis—the formation of new blood vessels from pre-existing vasculature—under both physiological and pathological conditions. HIF-1 regulates the expression of VEGF, a potent angiogenic factor, as well as other pro-angiogenic growth factors such as angiopoietin-2 and stem cell factor (SCF), contributing significantly to wound repair (77–79). Positive regulators of HIF-1, such as prolyl-4-hydroxylase inhibitors, have shown therapeutic potential in promoting the closure of diabetic ischemic wounds. HIF-1 deficiency under pathological conditions can lead to impaired or delayed wound healing (80–82).

Our data on apoptosis activation during wound healing are consistent with current scientific perspectives. Apoptosis has been previously described in a wide range of physiological processes; however, its role in skin wound healing has only recently begun to receive focused attention. Apoptosis is essential for normal wound healing, particularly for the removal of inflammatory cells and scar formation. As cell populations rapidly proliferate during tissue remodeling, cell growth is balanced by programmed cell death. For example, inflammatory cells must be cleared to allow progression to the next phase of wound healing; otherwise, persistent inflammation may result in chronic, non-healing wounds. Similarly, the cellularity of granulation tissue must be reduced as it transitions into scar tissue. Several key roles of apoptosis in wound healing have now been elucidated, including the apoptotic clearance of inflammatory cells (83–86). Apoptosis was consistently observed in inflammatory cells beneath the leading edge of the migrating epithelium. This may indicate that apoptosis serves as a signal marking the end of the inflammatory phase of wound healing. It is well established that pathological conditions such as diabetes and atherosclerosis can prolong apoptosis and negatively affect wound healing by suppressing the expression of the anti-apoptotic protein Bcl-2 (87, 88).

We found that chronic social stress prolonged apoptosis and led to a deficiency of Bcl-2. Our data also revealed changes in the SIRT1/Nrf2/p62 signaling pathway during wound healing under normal conditions and following chronic stress. The signaling pathway of nuclear factor erythroid 2–related factor 2 (NFE2L2 or NRF2) reduces oxidative damage and regulates the expression of antioxidant genes in response to oxidative stress, thereby enhancing cellular protection. Activation of the NRF2 pathway in-creases cellular resistance to chemical carcinogens and inflammation. This signaling cascade mediates anti-inflammatory and antioxidant effects by regulating calcium ions, mitochondrial oxidative stress, autophagy, ferroptosis, pyroptosis, and apoptosis. Following activation, NRF2 may also contribute to epithelial regeneration (89–93). Further studies have shown that activators or promoters of NRF2 can be effectively used to promote cellular healing (94). Elevated levels of NRF2 transiently modulate the expression of vascular-related genes in wounds, which may accelerate the healing of chronic wounds. However, the exact mechanism of NRF2 action remains unclear. It is known, however, that NRF2 primarily affects the inflammatory and proliferative phases of wound healing (95, 96).

We obtained interesting data indicating that the SIRT1/Nrf2/p62 signaling pathway is disrupted by chronic social stress. Stress can affect the progression through the different phases of wound healing via multiple immune and neuroendocrine path-ways. It has been established that stress-induced glucocorticoid production is associated with delayed wound healing. Both upregulation and downregulation of pro- and anti-inflammatory cytokines by glucocorticoids have been reported (11).

**Figure 2:**
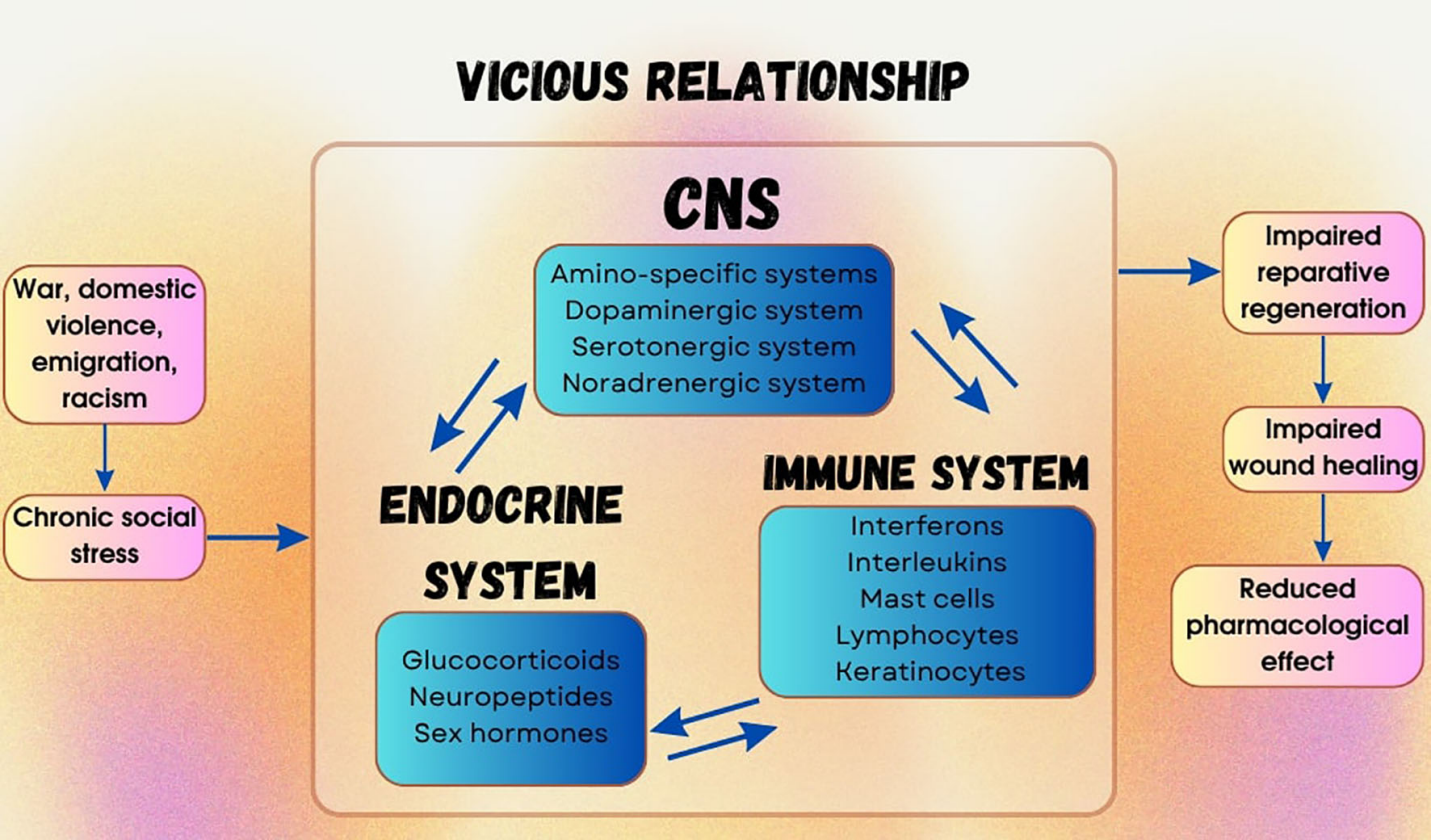
Pathological interconnection between chronic social stress and impaired wound healing. Chronic psycho-emotional and socially conflicted stress, along with other behavioral factors, exerts a detrimental impact on wound healing and significantly reduces the efficacy of reparative-type pharmacological agents. Chronic social stress initiates and sustains a pathological interplay between the central nervous, endocrine, and peripheral nervous systems, giving rise to neuro-immune-endocrine dysregulation that delays reparative regeneration of mechanical skin wounds. Prolonged exposure to adverse psycho-emotional environments or socially conflicting situations is perceived and processed by the brain, triggering a stress response via alterations in amino-specific pathways of the central nervous system. Through associated mediators, this negative signaling is transmitted to peripheral tissues, stimulating the release of hormones and neuropeptides. These molecules bind to their corresponding receptors on target cells through a cascade of intracellular signaling pathways. This stress-induced response activates the immune system, involving lymphocytes, mast cells, keratinocytes, and interleukins. The establishment of a vicious cycle ultimately results in persistent neuro-immune-endocrine imbalance, which impairs wound healing processes and reduces tissue regenerative capacity.

Chronic social stress leads to dysfunction of the brain’s aminospecific (monoaminergic) system. In a model of social defeat stress, decreased serotonin levels were observed along with mood and anxiety disorders. These changes were accompanied by reduced serotonin release, impaired reuptake, and decreased expression of the serotonin transporter protein (SERT) (97–99) (**Figure 2**).

Serotonin, or 5-hydroxytryptamine (5-HT), is a monoamine neurotransmitter best known for its antidepressant properties within the central nervous system (CNS). Serotonin elicits diverse cellular responses through various G protein–coupled serotonergic receptors (GPCRs), which are further classified into seven major families of serotonin receptors (5-HTR1 to 5-HTR7). Serotonin stimulation is known to play an important role in hemostasis and tissue repair (100, 101). The regenerative and fibroproliferative potential of serotonin has also been previously documented across various organ systems. Blockade of 5-HTR signaling impairs skin regeneration, while serotonin deficiency enhances apoptosis. Apoptosis also plays a significant role in burn wound progression (102, 103). Inhibition of apoptosis may represent a novel approach in the comprehensive therapy of wound progression. The role of serotonin has been investigated in studies using selective serotonin reuptake inhibitors (SSRIs) in animals with experimental wounds (104). Positive modulation of serotonin levels leads to increased expression of α-smooth muscle actin (α-SMA), which may indicate enhanced transdifferentiation of fibroblasts into myofibroblasts. Various cellular and matrix cytokines and growth factors, including connective tissue growth factor (CTGF), PDGF, TGF-β, matrix hyaluronan fragments, as well as physical factors such as mechanical tension, participate in maintaining the myofibroblast pool and may depend on serotonin levels (105). The chronic social stress model reproduced in our study, based on the identified specific impairments in animals’ psycho-emotional behavior (anxiety, fear, depression), was accompanied by serotonin deficiency. All these findings allow us to hypothesize that the negative effects of chronic social stress on skin reparative regeneration processes may be associated with suppressed serotonergic transmission and serotonin deficiency.

It has been established that social stress leads to suppression of dopaminergic transmission in the mesolimbic, tuberoinfundibular, and mesocortical systems of the brain. Chronic psychosocial adversity induces dopaminergic dysfunction. Prolonged exposure to psychosocial stressors (social isolation, experiences of racial and social discrimination, victimization, and social defeat) is associated with a significant reduction in dopamine synthesis capacity in the striatum, particularly in the limbic (ventral) striatum (106–109).

Dopamine is an important catecholaminergic neurotransmitter released by sympathetic nerve endings, and recent studies have demonstrated its potent anti-angiogenic effects mediated through its receptors. Dopamine plays a role in the neovascularization of dermal wound tissue and consequently in the wound healing process. Dopamine regulates the expression of the homeobox transcription factor HoxD3 and its target integrin α5β1, both of which play key roles in wound angiogenesis (110–113). Stimulation of dopamine receptors present in dermal fibroblasts restores the production of vascular endothelial growth factor A (VEGF-A) by these cells, leading to adequate angiogenesis and subsequent skin wound healing. This effect of dopamine D1 receptors is mediated through the protein kinase A (PKA) signaling pathway (114).

Dopamine deficiency leads to impaired expression of this factor and disrupts the wound healing process. Dopamine plays a critical role in angiogenesis and wound repair by regulating various pro-angiogenic factors, such as VEGF, to support the growth, survival, and differentiation of endothelial cells (113, 115, 116). High concentrations of dopamine can lead to increased phosphorylation of VEGFR-2 and Akt (serine/threonine-protein kinase) and promote migration of human mesenchymal progenitor cells (hMPCs) through activation of D2 receptors on the surface of hMPCs. Dopamine may enhance angiogenesis and wound healing via activation of D1 receptors, while simultaneously impairing wound healing when D2 receptors are activated (117–120).

Analysis of the data from this study suggests that the chronic social stress model we employed—based on behavioral changes such as reduced general locomotor activity, decreased exploratory behavior, and less time spent in illuminated areas of the open field test—indicates significant suppression of dopaminergic transmission, which is also associated with impaired wound healing.

Social stress may lead to long-lasting disruptions in the nitric oxide signaling system. It has been shown that post-weaning social isolation results in altered expression of nitric oxide synthase isoforms, including decreased expression of neuronal NOS (nNOS) in the cerebral cortex and cerebellum, and increased inducible NOS (iNOS) expression in the hippocampus. This elevated oxidative state reduces nitric oxide (NO) bioavailability and promotes the formation of peroxynitrite (121–123). We do not have direct or indirect experimental data regarding changes in the nitric oxide (NO) system in our rats following chronic social stress. However, based on the aforementioned studies, such assumptions can reasonably be made. It is known that NO and its cytotoxic derivatives increase cellular sensitivity to Fas receptor–mediated signaling. Peroxynitrite can suppress the expression of the anti-apoptotic protein Bcl-2 (122) Nitrosative stress delays angiogenesis, suppresses transcriptional activity, and impairs wound healing (123–125). Peroxynitrite negatively affects key physiological processes in the skin, including keratinocyte and fibroblast proliferation, collagen synthesis, microcirculation, innervation, and the immune response during wound healing (126). Additionally, researchers provided insight into the impact of psychological stress on neocollagenesis and scar formation, revealing implications for both tissue regeneration and cosmetic outcomes (127). ROS, NO, and its cytotoxic derivatives, in pathological conditions associated with impaired wound healing—such as stress, diabetes mellitus, atherosclerosis, and malignant tumors—disrupt the tissue growth factor TGF-α1 signaling pathway. They suppress the expression of key growth and repair factors, including FGF, HIF-1α, HSP70, and VEGF, leading to impaired angiogenesis and uncontrolled enhancement of apoptosis (128–132).

Emerging evidence highlights the systemic interplay between chronic stress, immune dysregulation, and metabolic disturbances mediated by transcriptional and microbial mechanisms (133, 134). Altered gut microbiota composition and short-chain fatty acid signaling influence neuroimmune regulation and may offer therapeutic perspectives for posttraumatic stress disorder and depression (135–138). Stress-induced transcriptional shifts affecting apoptosis, neurogenesis, and immune response have been observed in thyroid, endometrial, and renal disorders, emphasizing the role of genetic and epigenetic regulation in systemic stress vulnerability (139–145). Furthermore, modulation of nuclear receptors such as AHR, FFAR2, FXR, and TGR5 links metabolic-associated fatty liver disease, COVID-19, and oxidative stress (146–148). These findings suggest convergent molecular pathways underlying inflammation, stress, and tissue repair failure, pointing to potential benefits of metabolic modulators in comorbid disorders (149–152). Such an integrative understanding of stress-induced metabolic, immune, and transcriptional alterations may ultimately pave the way for personalized therapeutic strategies aimed at restoring systemic homeostasis and improving both mental and physical recovery.

## 5 Limitations of the study

This study has several methodological and translational limitations that should be acknowledged.

A primary limitation is the absence of analyses of the cellular response and transcriptional activity following social stress and wound induction, which prevents a deeper understanding of the intimate molecular mechanisms of wound healing under social stress conditions.

Another constraint is the limited temporal scope of the study. The effects of more prolonged or delayed mechanisms of social stress on wound repair and reparative regeneration, including the influence of prenatal social stress, were not investigated.

The use of a single animal model represents an additional limitation. Employing only a model of social stress restricts generalization to other types of cognitive or neurobehavioral disturbances and their potential effects on wound healing. Validation of findings in additional models—such as traumatic brain injury, ischemia, aging, post-traumatic stress disorder (PTSD), barotrauma, or emotional stress—would strengthen translational relevance.

The study also used a relatively small sample size (n = 6 per group). While sufficient to detect major effects, this number may be underpowered to identify subtle mechanisms or weak associations.

From a mechanistic perspective, receptor–ligand interactions predicted by molecular docking were not directly validated using experimental receptor-binding assays, leaving uncertainty about the actual molecular targets involved. Moreover, neurobiochemical and molecular analyses did not include all relevant markers necessary for a comprehensive assessment of the pathways underlying anxiety and altered neuroplasticity induced by social stress.

Translationally, several additional factors may limit generalization. The study included only adult rats (12–13 months old), and only males were examined, which limits the assessment of potential sex differences in response to social stress and its impact on wound healing, thereby restricting generalizability. The exclusion of females also prevents evaluation of the influence of testosterone and estrogen levels on susceptibility or resilience to social stress and on the molecular mechanisms of wound healing, further limiting the ability to generalize the findings. Behavioral assessment was limited mainly to the open field test. Inclusion of additional cognitive tasks focused on learning and memory—such as the Morris water maze or novel object recognition—would have provided stronger evidence for links between cognitive function, stress resilience, and wound-healing mechanisms.

Finally, species-related and age-related limitations should be noted. The exclusive use of rats restricts direct extrapolation to humans without further validation in other species or human-derived cellular models. Moreover, only adult rats (12–13 months old) were studied, leaving unanswered questions regarding the effects of social stress on wound healing in developing or aging organisms.

Future studies aimed at investigating the impact of PTSD and social stress on wound healing will address these limitations. Planned work will also explore the effects of geroprotective agents, nootropics, and antidepressants on enhancing baseline reparative therapy.

## 6 Conclusion

Our results revealed the detrimental impact of chronic social stress on the skin wound healing process. Apparently, neurochemical, hormonal, and other stress-induced disturbances contribute to enhanced and prolonged apoptosis, reduced transcriptional activity, and decreased expression of endogenous cytoprotective factors—ultimately leading to impaired healing of mechanical skin wounds.

These findings are not only of fundamental importance and help clarify the molecular mechanisms underlying wound repair, but also suggest new potential targets for pharmacological modulation of skin regenerative processes following injury.

## Data Availability

The raw data supporting the conclusions of this article will be made available by the authors, without undue reservation.
