# Supplementary material for: Impact of chronic social stress on molecular markers of skin regeneration during experimental excisional wounding
Source: Front Immunol. 2025 Dec 3;16:1656214. doi: 10.3389/fimmu.2025.1656214 (PMC12708941; doi:10.3389/fimmu.2025.1656214)
Supplement: Supplementary file 1 [file DataSheet1.pdf]

Supplementary Material

Supplementary Material for

Impact of Chronic Social Stress on Molecular Markers of Skin Regeneration During Experimental Excisional Wounding

Supplementary Figures: 1 to 4

Legends for Supplementary Figures: 1 to 4

Supplementary Material

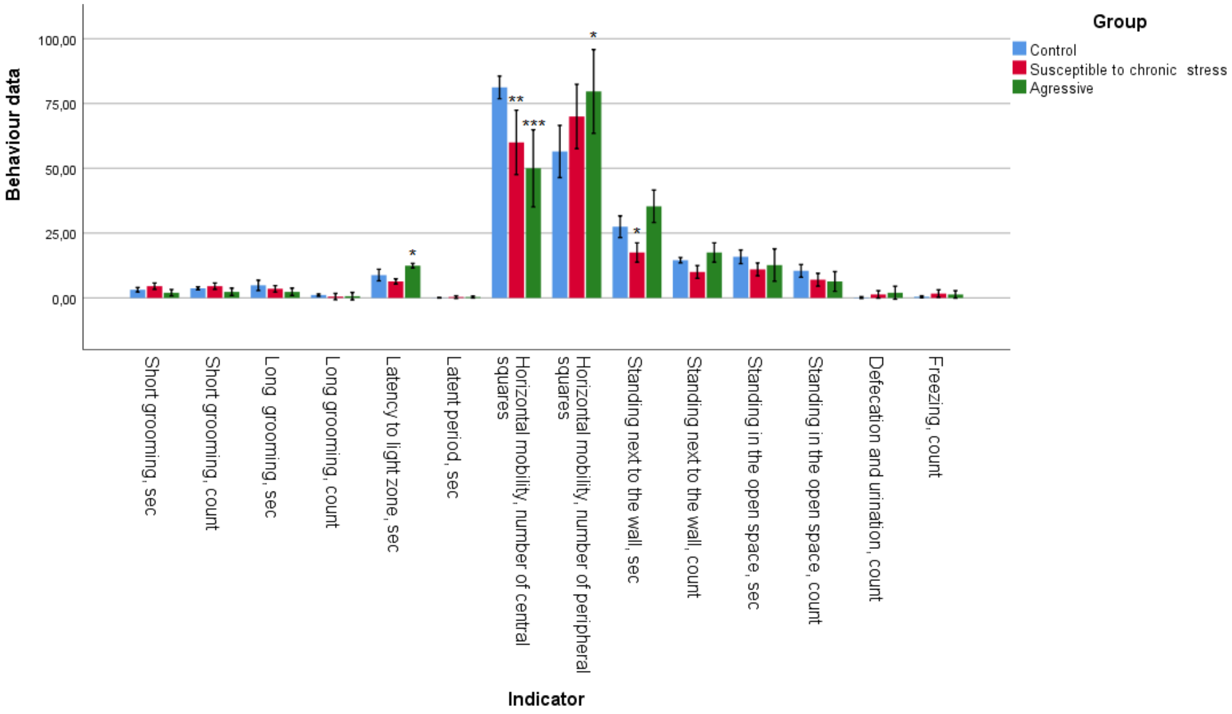

A

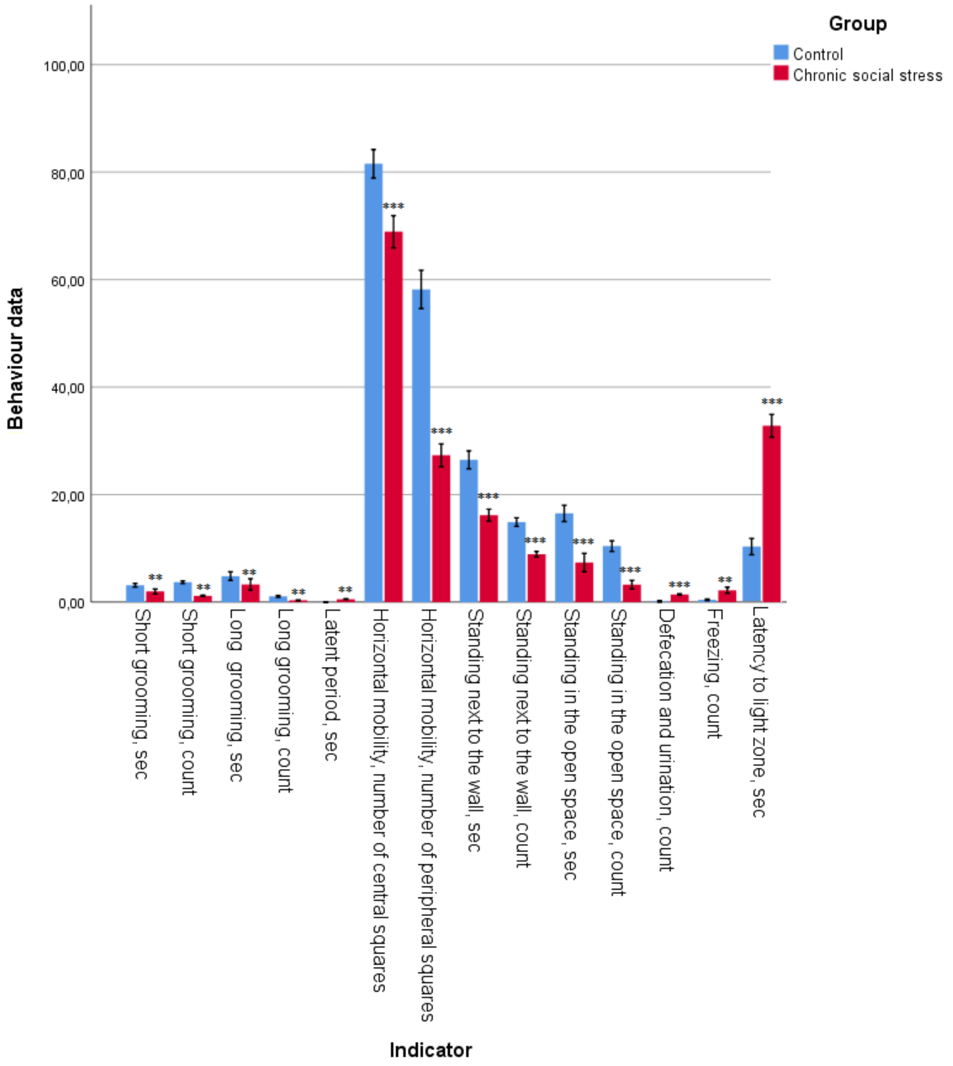

B

Supplementary Figure 1. The "Open field" data. (A) Prior to stressing and (B) after 3-week social stress. Data represent the mean ± SD with significance compared to control determined by Student's t-test; *p<0.05; **p<0.01; ***p<0.001; ****p<0.0001.

Supplementary Material

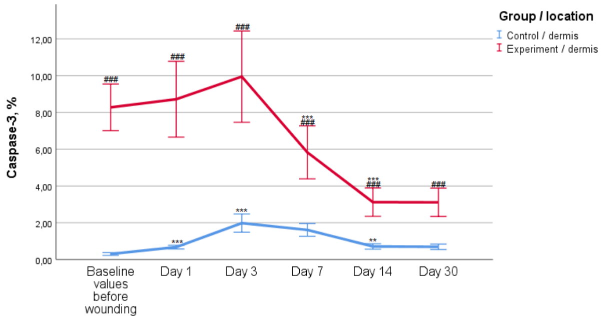

A

B

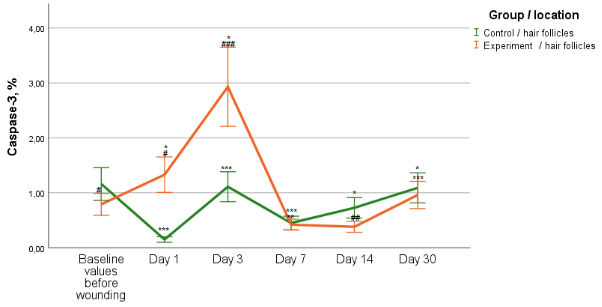

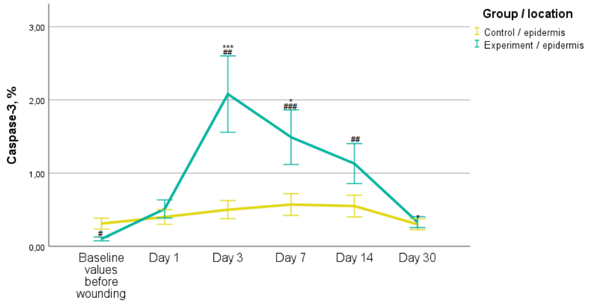

C

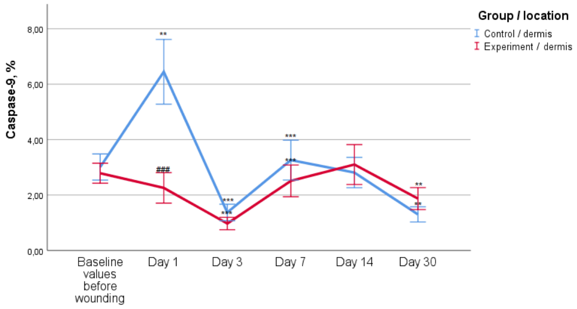

D

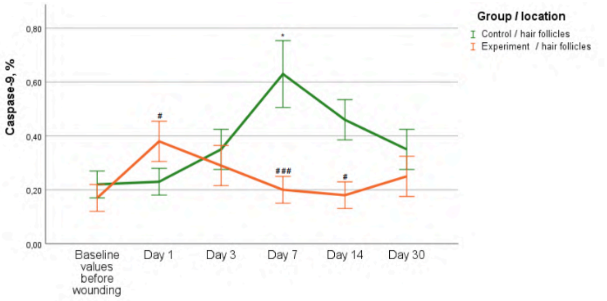

E

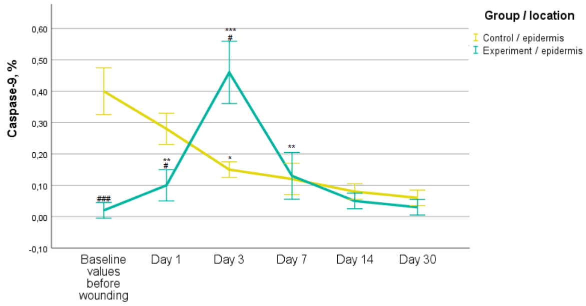

F

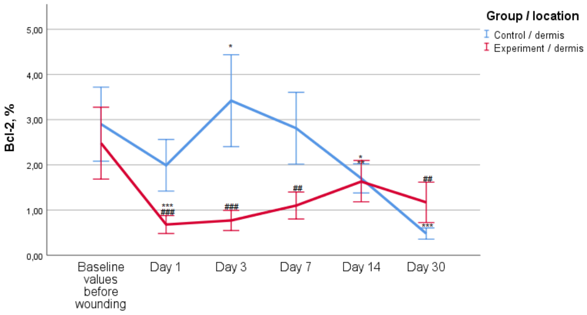

G

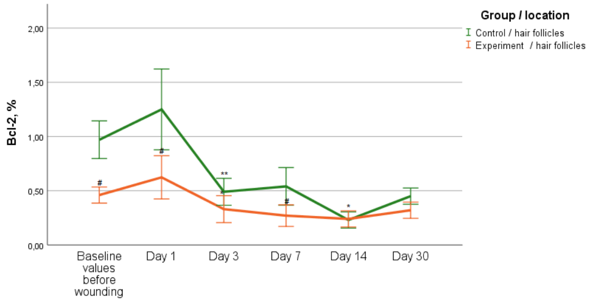

H

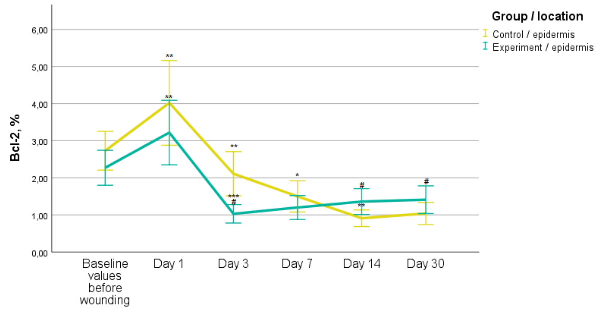

I

Supplementary Figure 2. Dynamics of apoptosis marker expression (Caspase 3 (A-C), Caspase 9 (D-F) and Bcl-2 (G-I)) in different skin tissues of rats at various time points after wounding. The data correspond to the mean ± SD; * p<0.05, ** p<0.01, ***p<0.001 compared to the previous period of wound healing; #p<0.05, ## p<0.01, ### p<0.001 changes are statistically significant, compared to control data at the same time period of wound healing

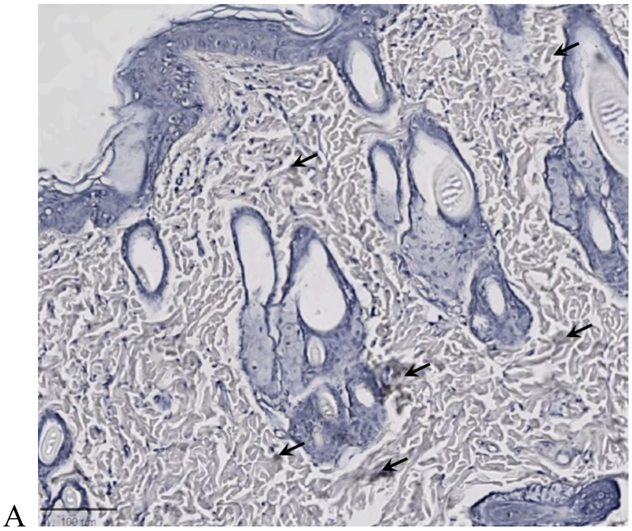

A

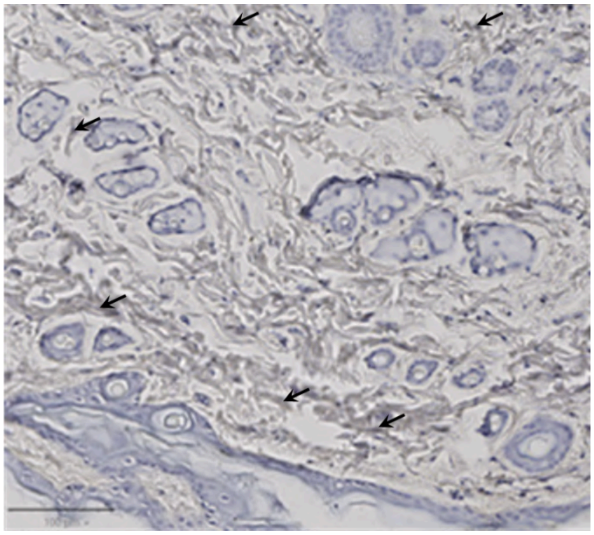

B

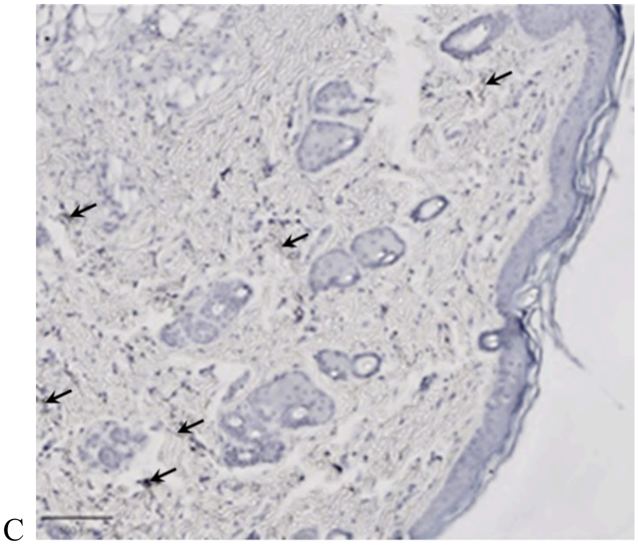

C

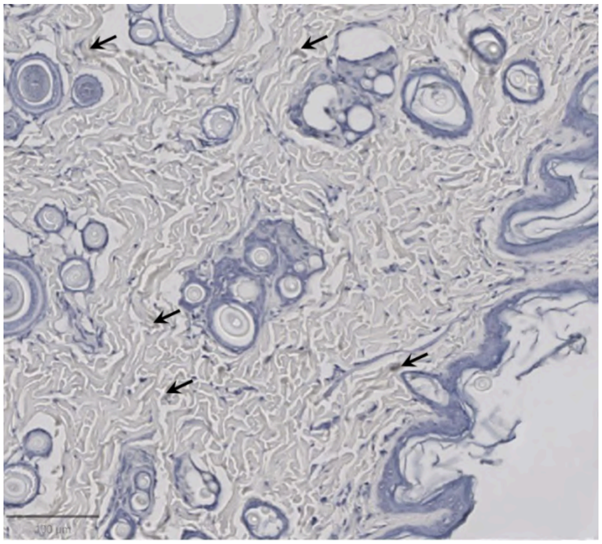

D

Supplementary Material

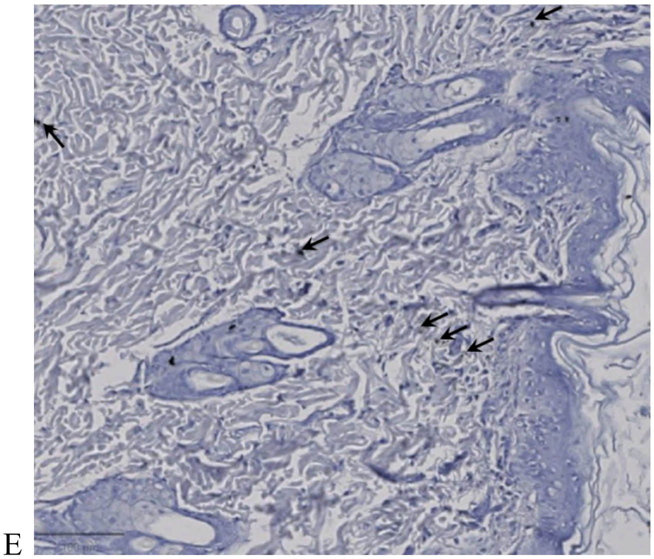

E F

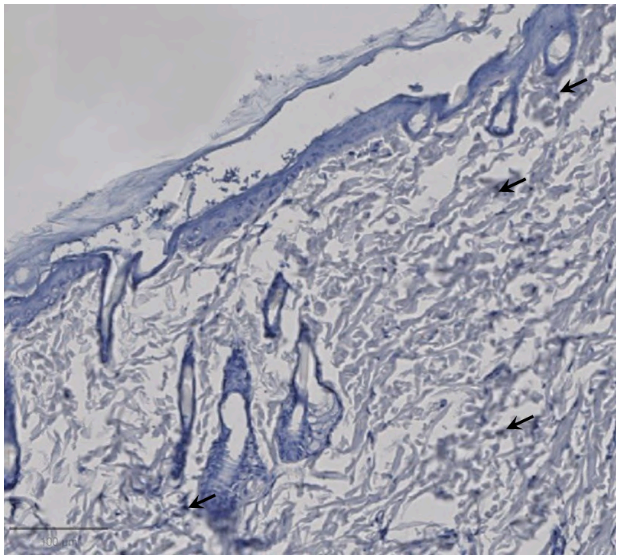

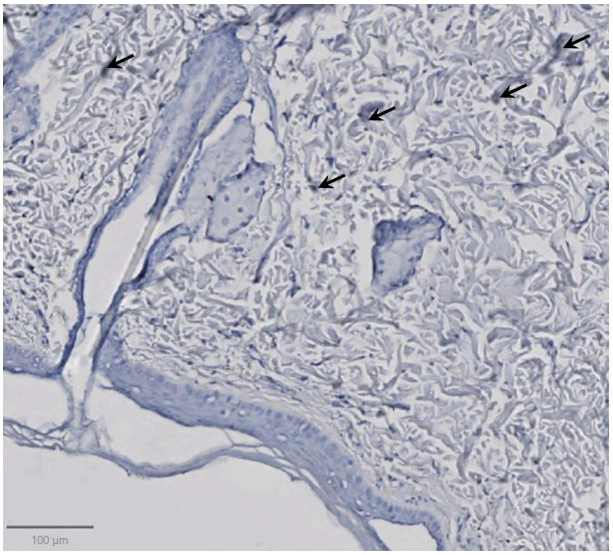

G

H

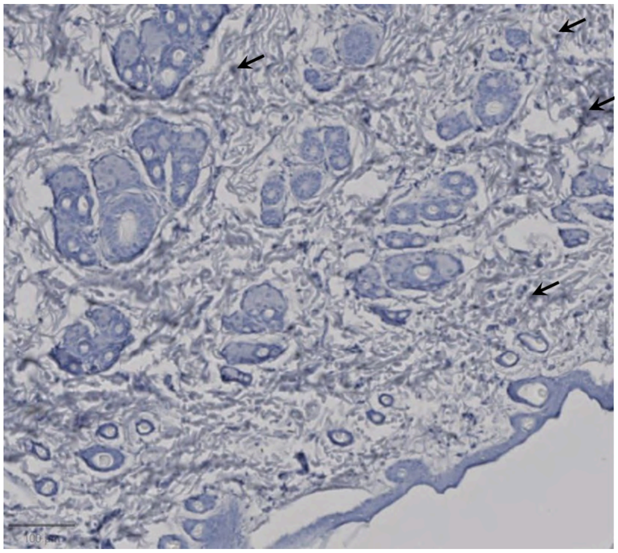

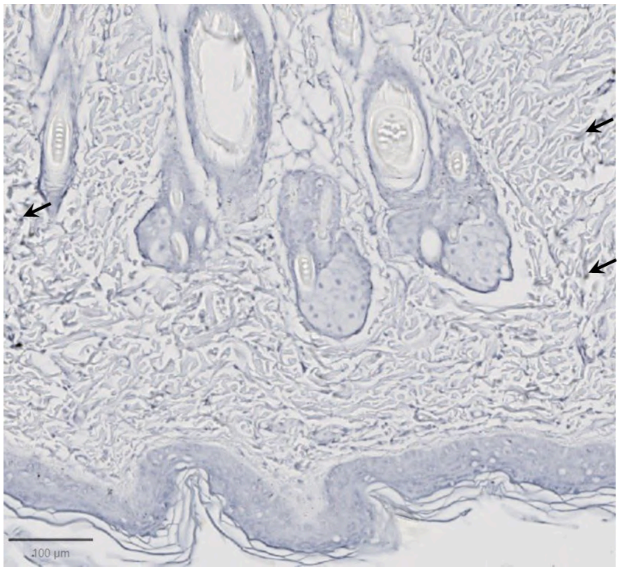

I J

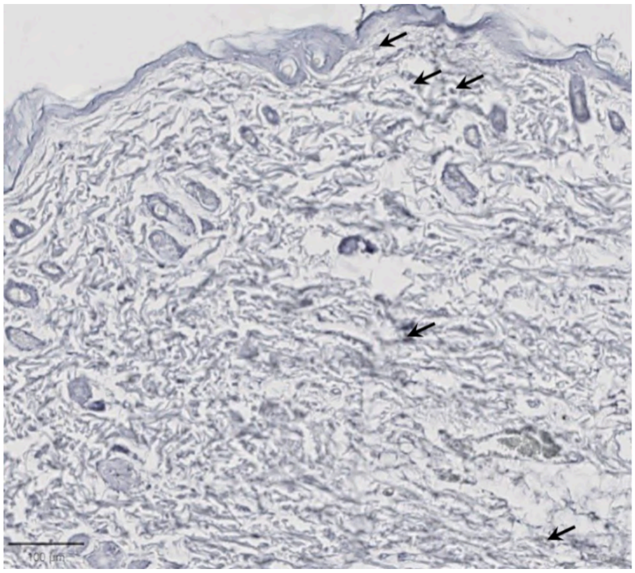

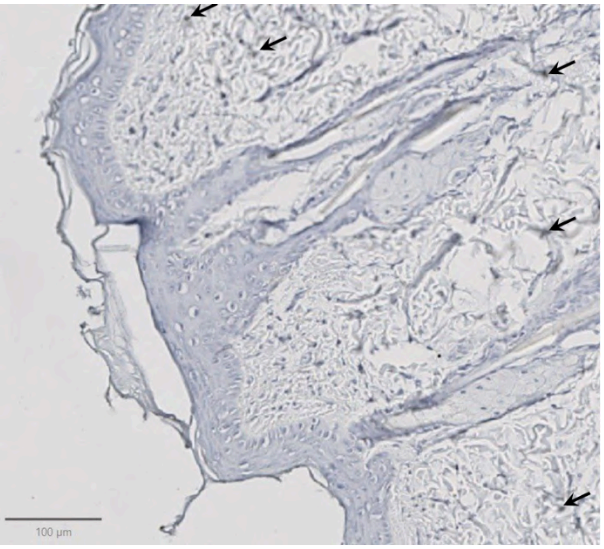

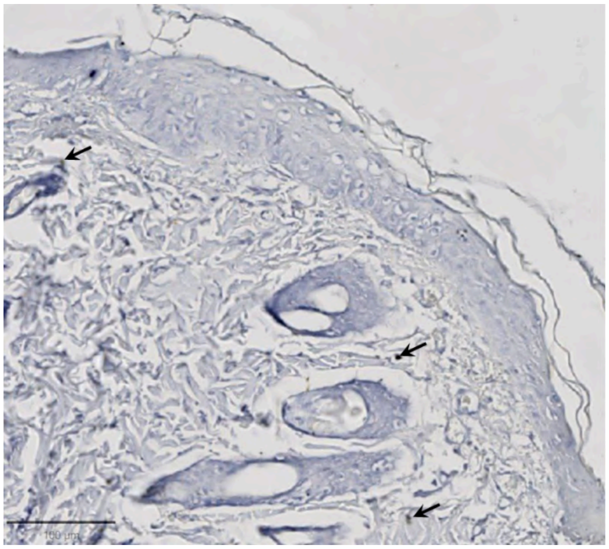

K L

Supplementary Figure 3. Apoptosis markers expression (IHC with DAB) in rat skin at various time points after wounding in control and experimental groups. (A-D) Caspase 3 expression prior to wounding in control (A) and experimental groups (B), on day 3 of wound healing in control (C) and experimental groups (D). (E-H) Caspase 9 expression prior to wounding in control (E) and experimental groups (F), on day 3 of wound healing in control (G) and experimental groups (H). (I-L). Bcl-2 expression prior to wounding in control (I) and experimental groups (J), on day 3 of wound healing in control (K) and experimental groups (L). Some of the marked cells are pointed with arrows.

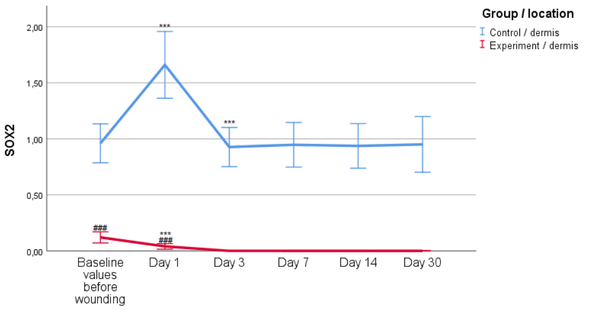

A B

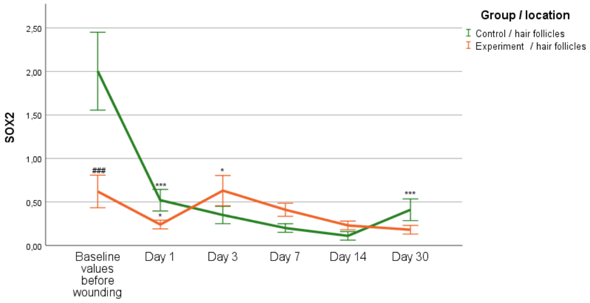

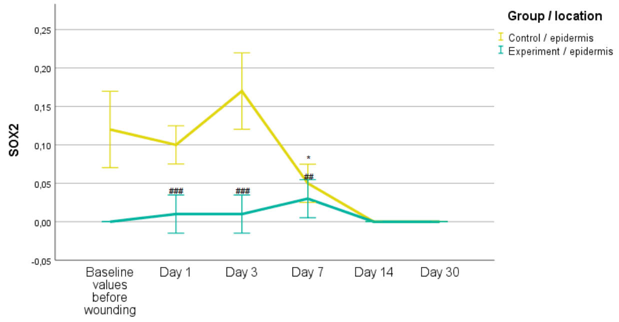

C D

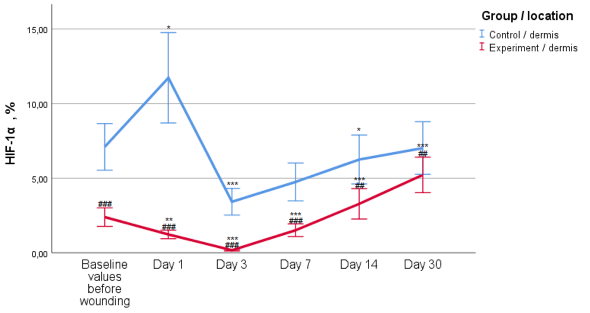

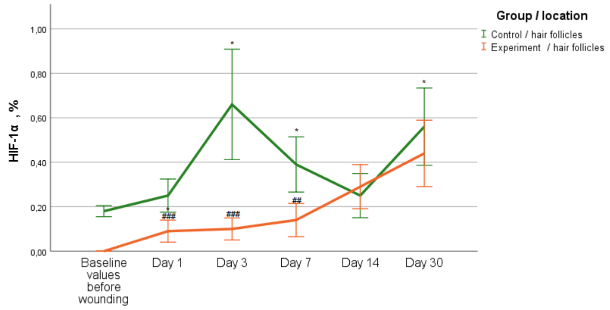

E

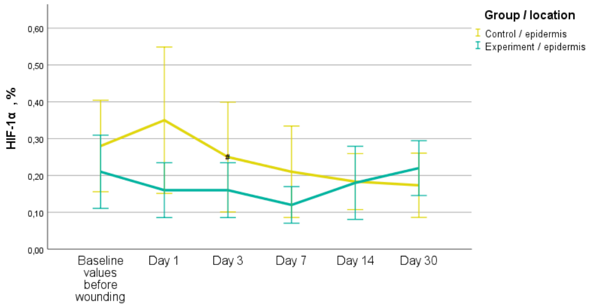

F

Supplementary Figure 4. Dynamics of changes in endogenous cytoprotection markers (SOX2 (A-C), HIF-1α (D-F)) expression in different skin tissues of rats at various time points after wounding. The data correspond to the mean ± SD; * p<0.05, ** p<0.01, ***p<0.001 compared to the previous period of wound healing; #p<0.05, ## p<0.01, ### p<0.001 changes are statistically significant, compared to control data at the same time period of wound healing.

A

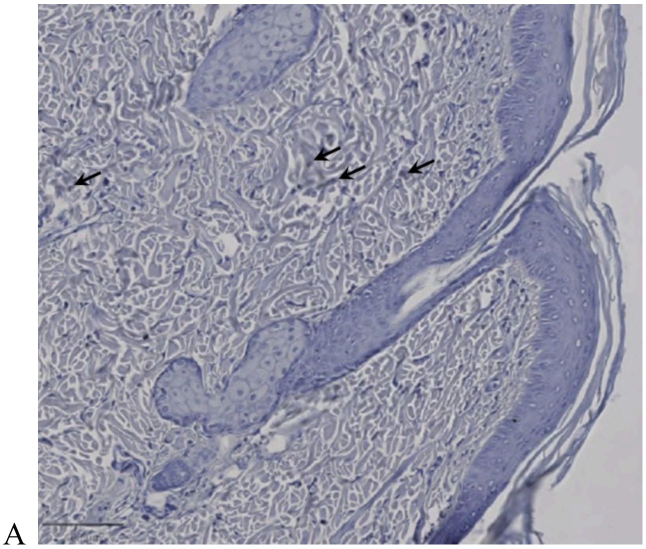

B

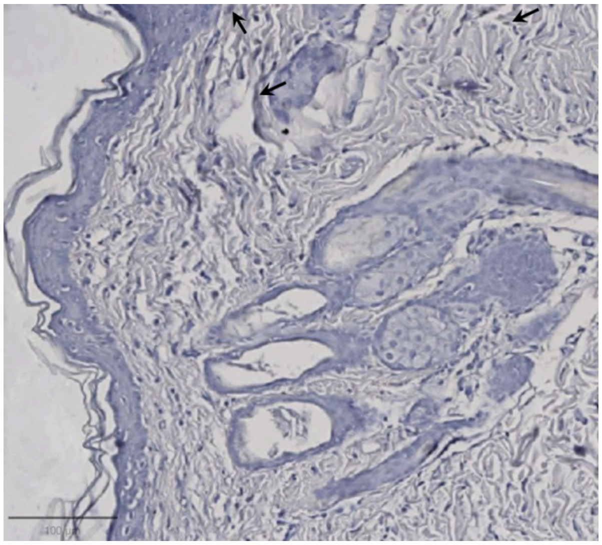

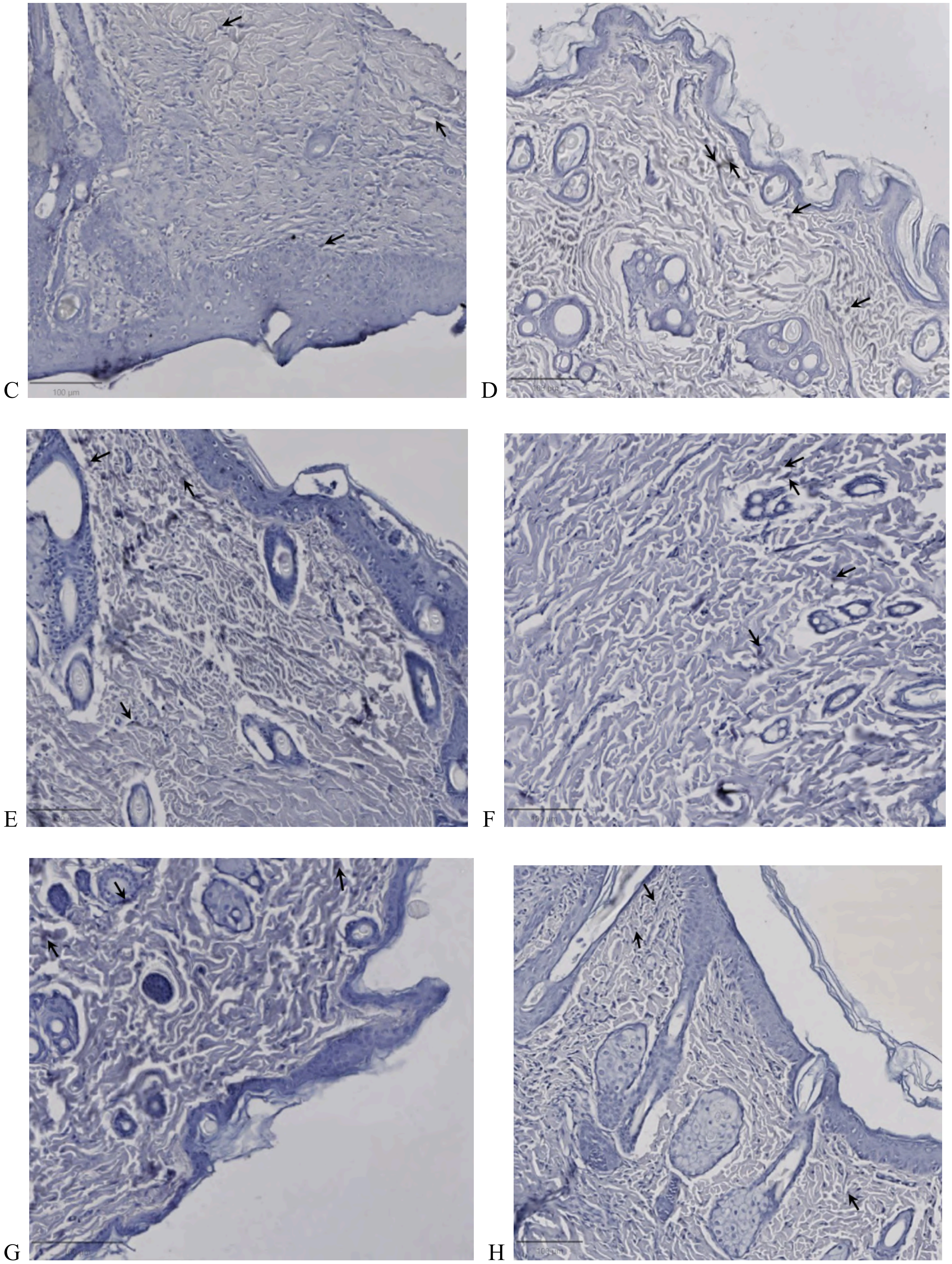
C
D
E
F
G
H
100 µm

Supplementary Material

Supplementary Figure 5. Changes in endogenous cytoprotection markers expression (IHC with DAB) in rat skin at various time points after wounding in control and experimental groups. (A-D) SOX2 expression prior to wounding in control (A) and experimental groups (B), on day 3 of wound healing in control (C) and experimental groups (D). (E-H) HIF-1α expression prior to wounding in control (E) and experimental groups (F), on day 3 of wound healing in control (G) and experimental groups (H). Some of the marked cells are pointed with arrows.

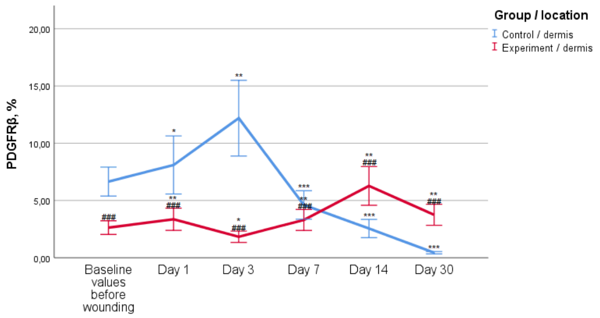

A

B

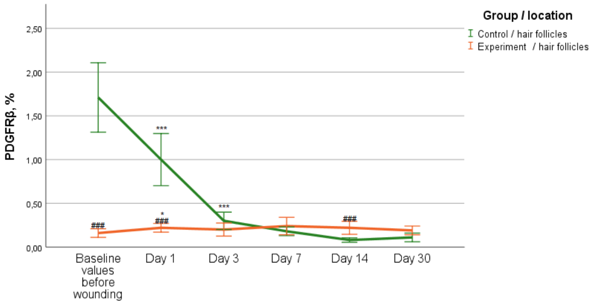

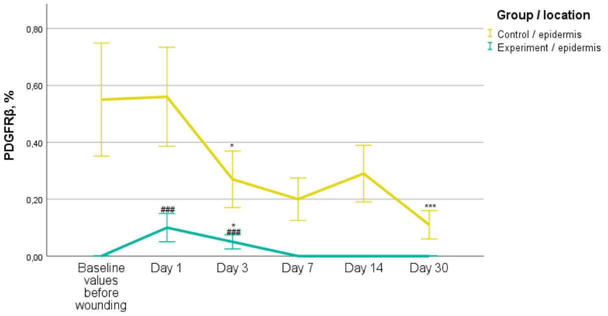

C

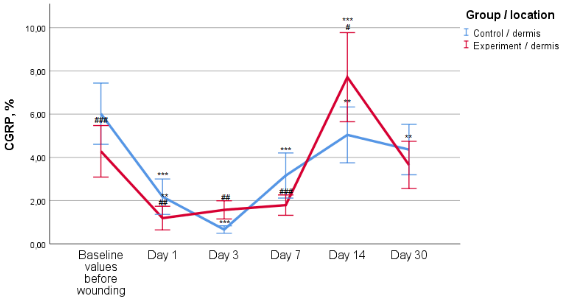

D

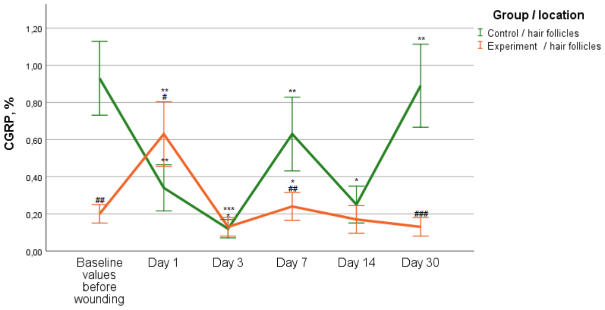
CGRP, %
Group / location
Control / hair follicles
Experiment / hair follicles
Baseline values before wounding
Day 1
Day 3
Day 7
Day 14
Day 30

E F

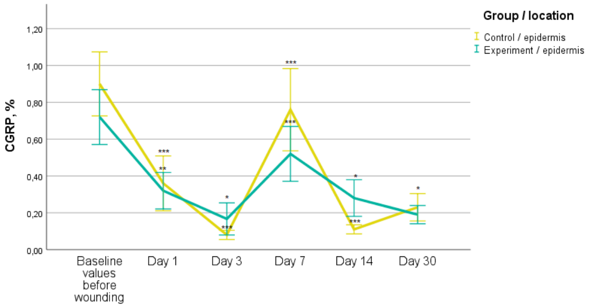
CGRP, %
Group / location
Control / epidermis
Experiment / epidermis
Baseline values before wounding
Day 1
Day 3
Day 7
Day 14
Day 30

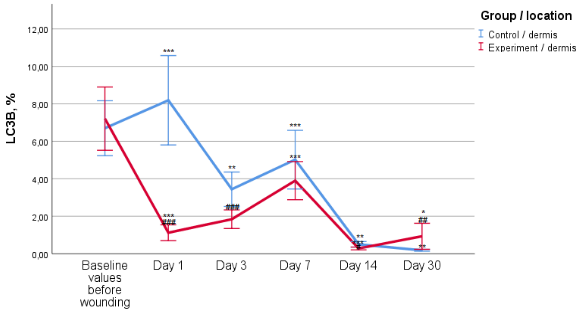
LC3B, %
Group / location
Control / dermis
Experiment / dermis
Baseline values before wounding
Day 1
Day 3
Day 7
Day 14
Day 30

G H

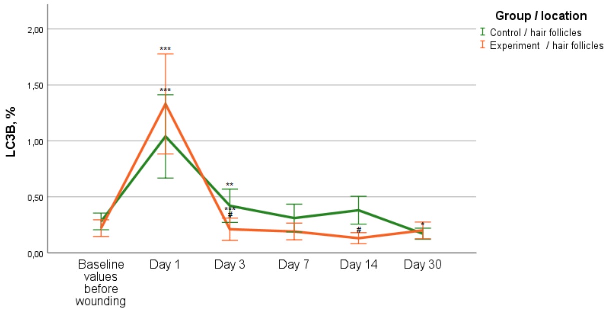
LC3B, %
Group / location
Control / hair follicles
Experiment / hair follicles
Baseline values before wounding
Day 1
Day 3
Day 7
Day 14
Day 30

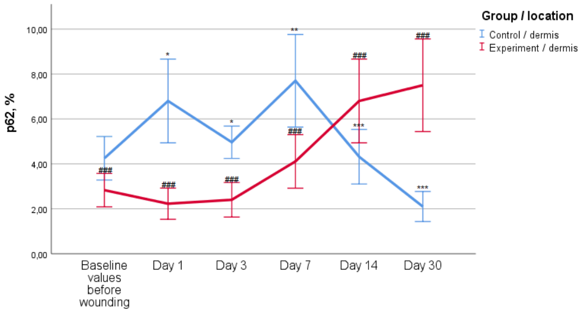
p62, %
Group / location
Control / dermis
Experiment / dermis
Baseline values before wounding
Day 1
Day 3
Day 7
Day 14
Day 30

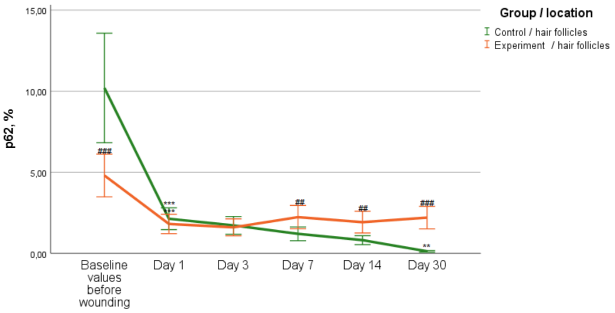
p62, %
Group / location
Control / hair follicles
Experiment / hair follicles
Baseline values before wounding
Day 1
Day 3
Day 7
Day 14
Day 30

I J

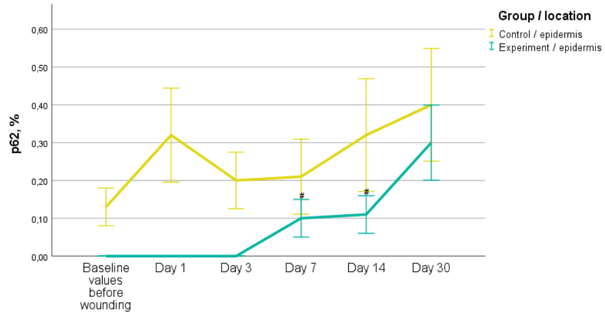

K

L

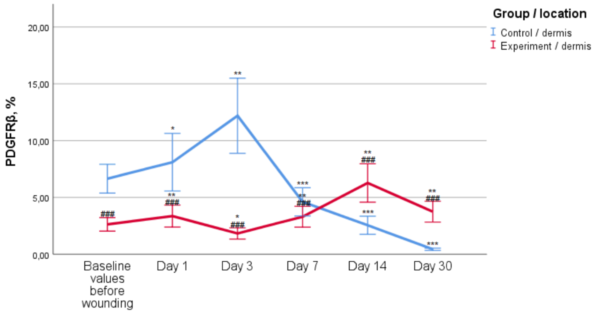

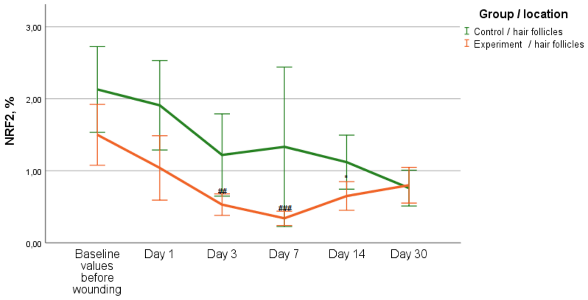

M

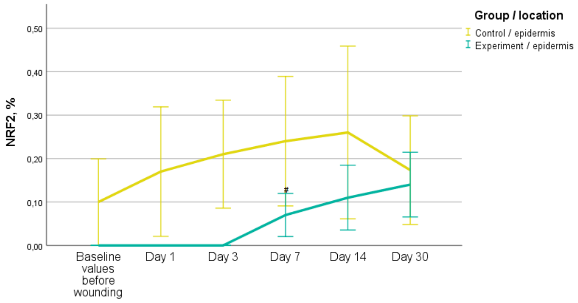

N

Supplementary Figure 6. Dynamics of transcription factor (PDGFRβ (A-C), CGRP (D-F), LC3B (G-H, not detected in epidermis), p62 (I-K), NRF2 (L-N)) changes expression in different skin tissues of rats at various time points after wounding. The data correspond to the mean ± SD; * $p<0.05$, ** $p<0.01$, ***$p<0.001$ compared to the previous period of wound healing; #$p<0.05$, ## $p<0.01$, ### $p<0.001$ changes are statistically significant, compared to control data at the same time period of wound healing.

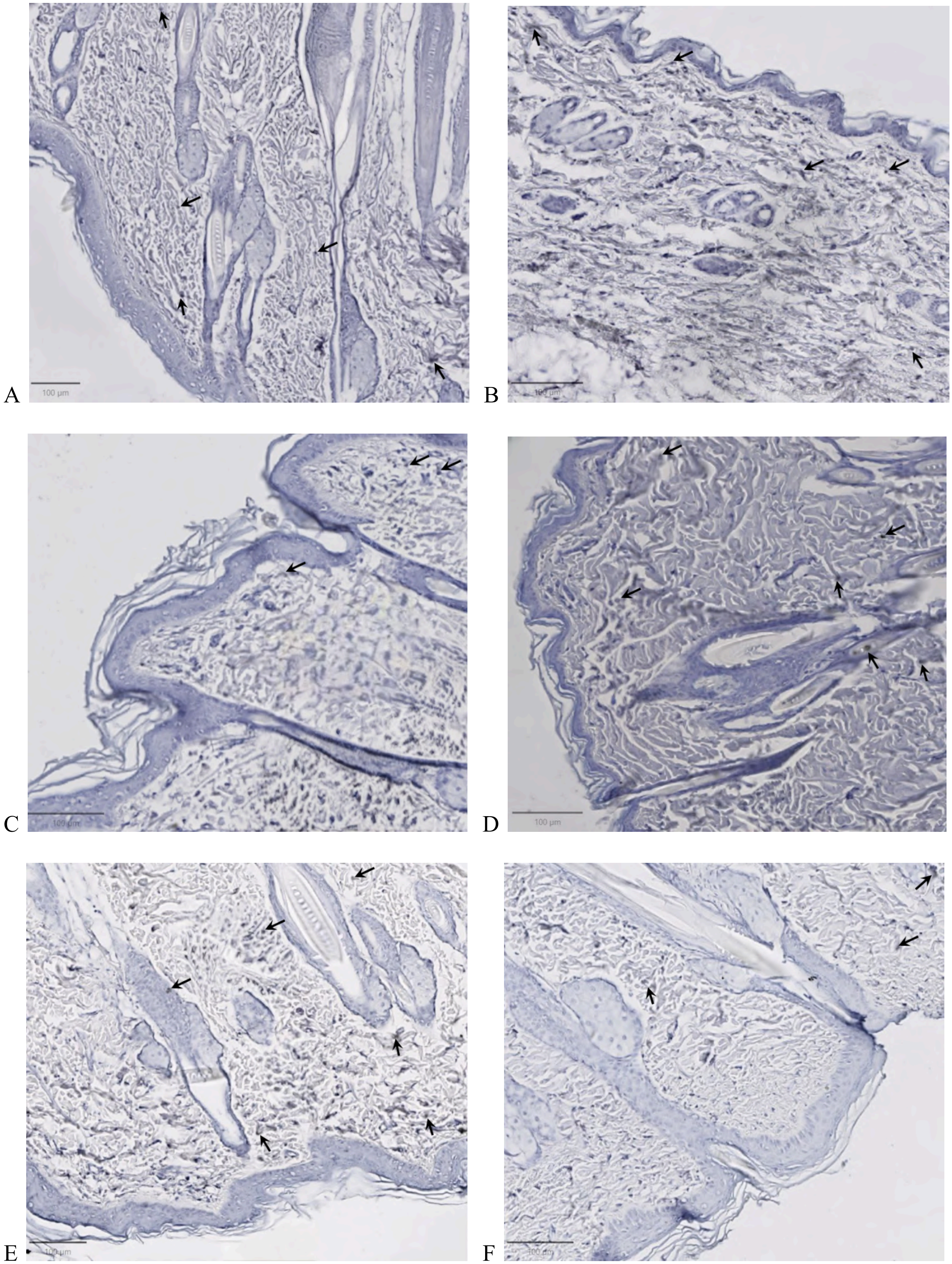
A
B
C
D
E
F
100 µm

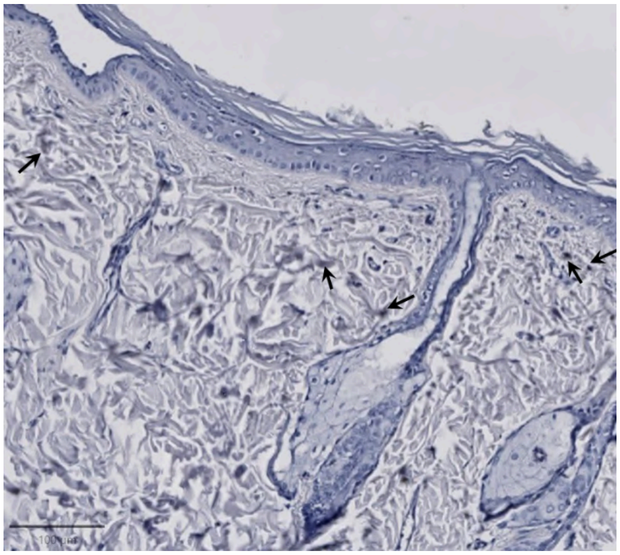

G

H

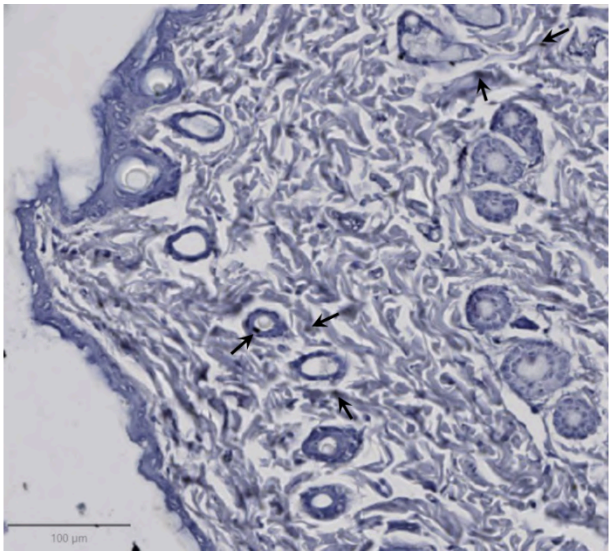

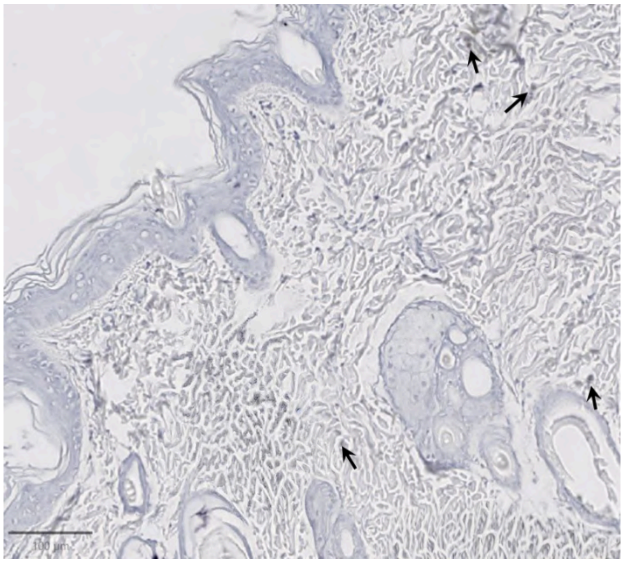

I

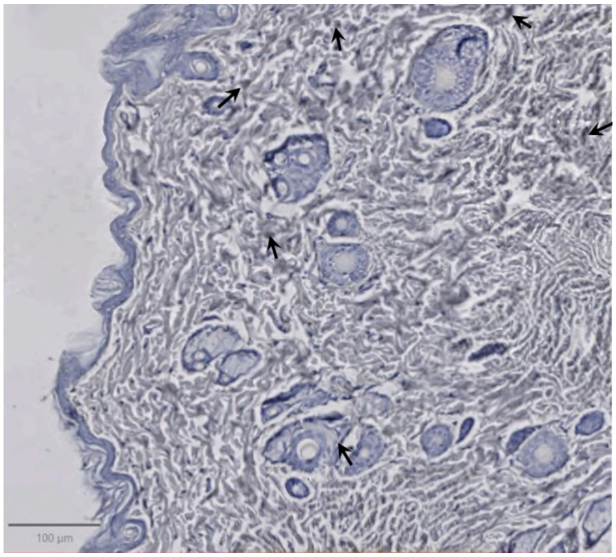

J

K 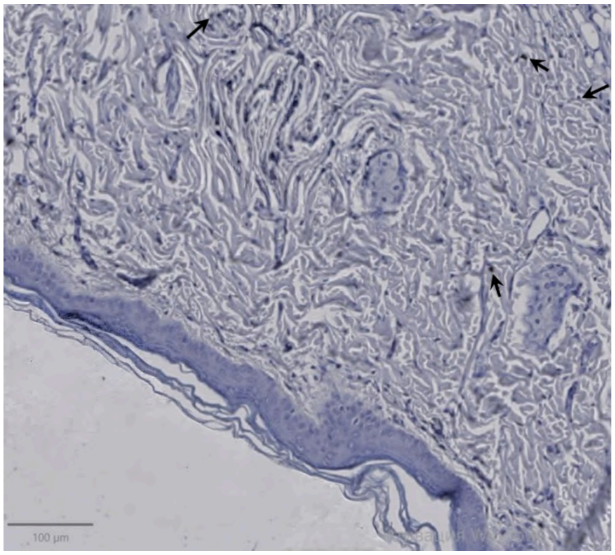

L 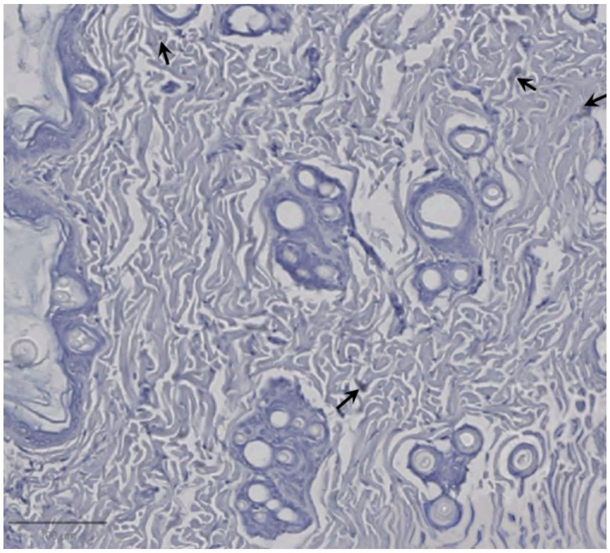

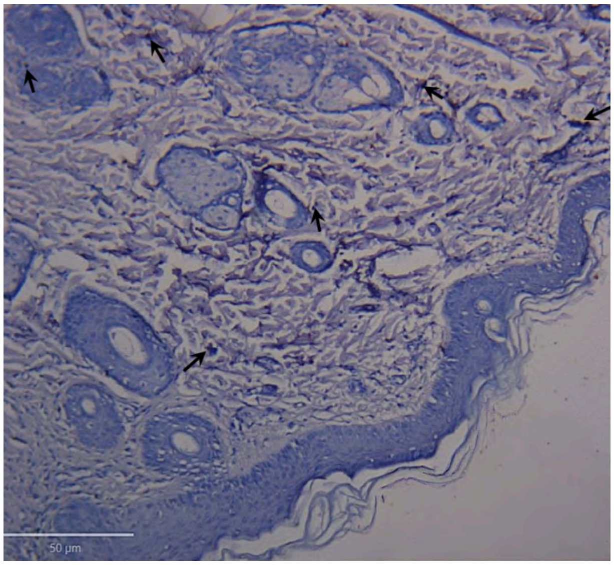

M

N

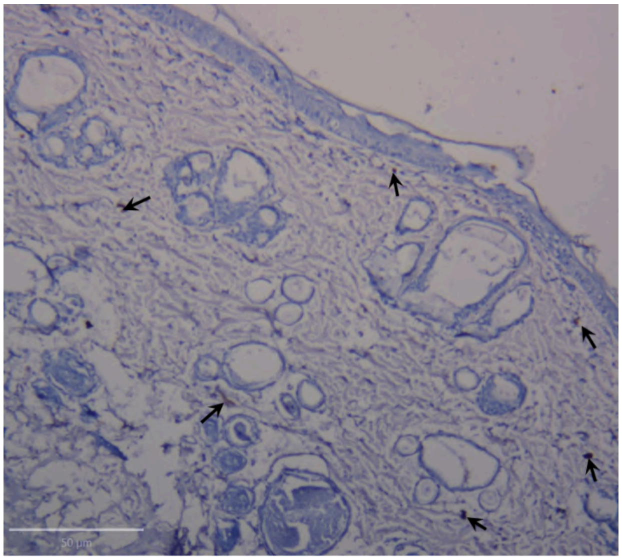

Supplementary Material

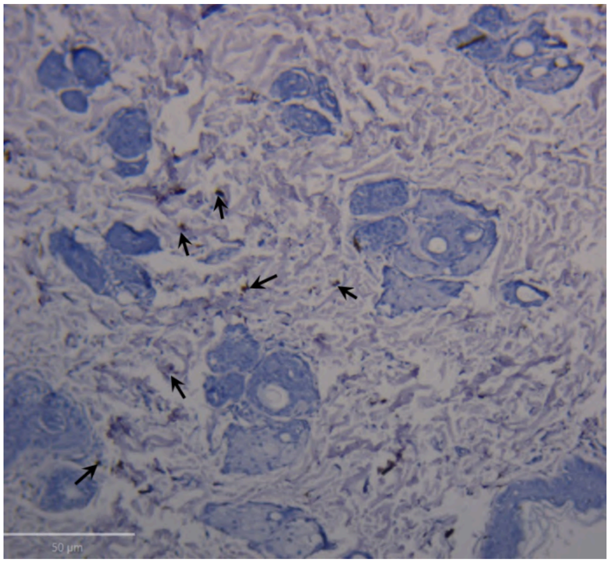

O P

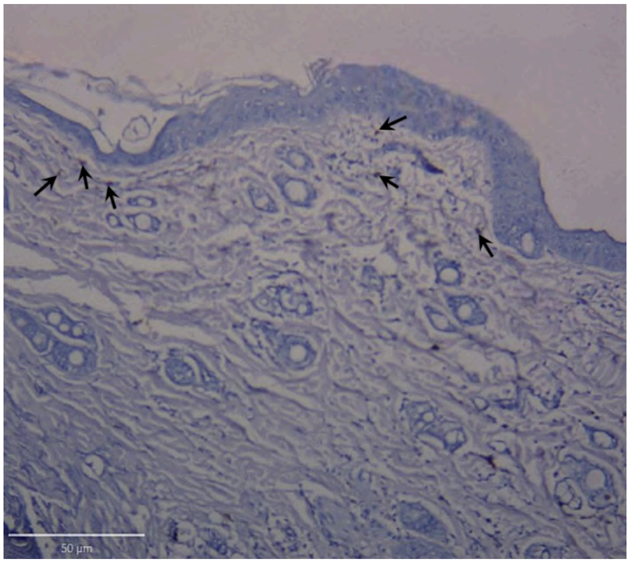

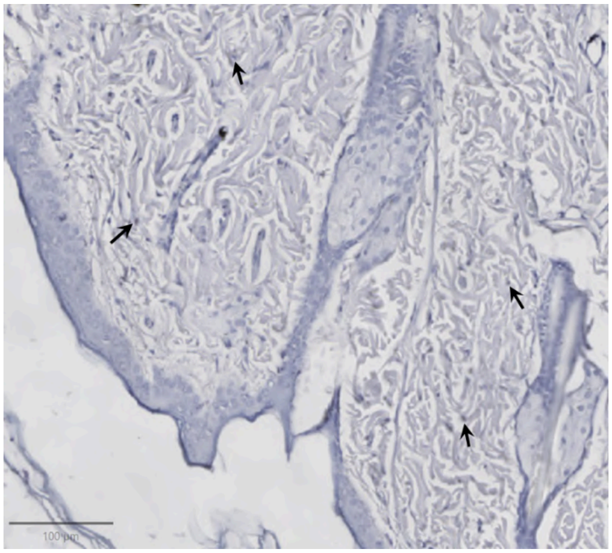

Q

R

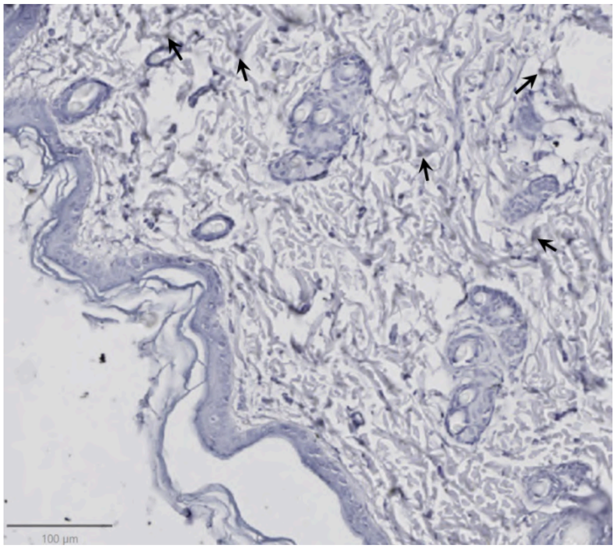

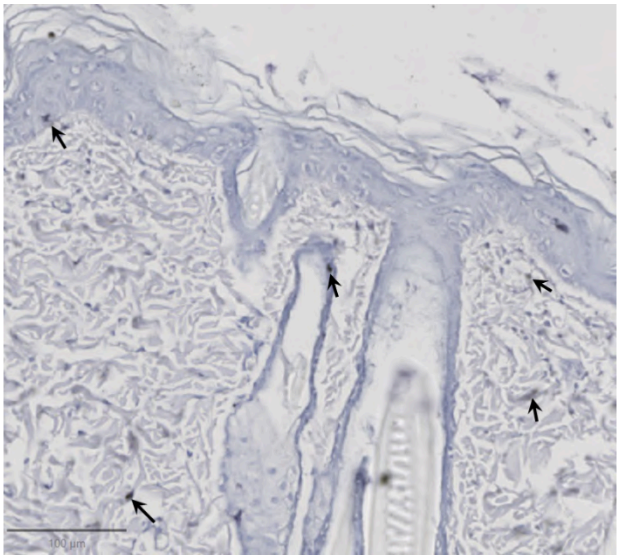

S T

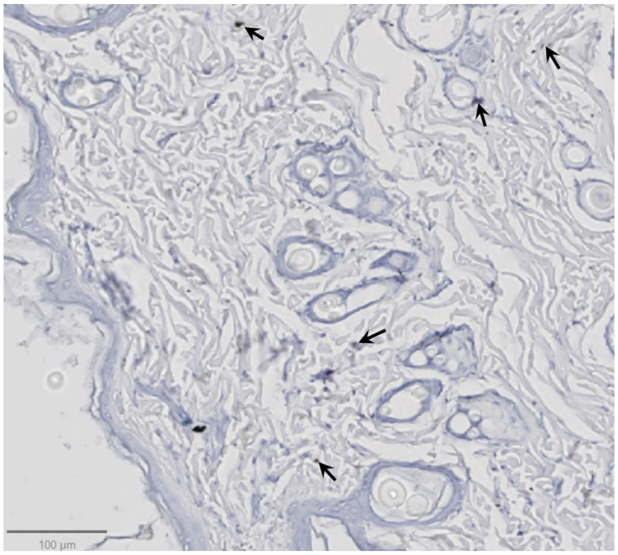

Supplementary Figure 7. Transcription factor expression (IHC with DAB) in rat skin at various time points after wounding in control and experimental groups. (A-D) PDGFRβ expression prior to wounding in control (A) and experimental groups (B), on day 3 of wound healing in control (C) and experimental groups (D). (E-H) CGRP expression prior to wounding in control (E) and experimental groups (F), on day 3 of wound healing in control (G) and experimental groups (H). (I-L). LC3B expression prior to wounding in control (I) and experimental groups (J), on day 3 of wound healing in control (K) and experimental groups (L). (M-P) p62 expression prior to wounding in control (M) and experimental groups (N), on day 3 of wound healing in control (O) and experimental groups (P). (Q-T). NRF2 expression prior to wounding in control (Q) and experimental groups (R), on day 3 of wound healing in control (S) and experimental groups (T). Some of the marked cells are pointed with arrows.
